# Susceptibility to mild and severe acute mountain sickness is associated with distinct urine metabolite profiles

**DOI:** 10.3389/fphys.2026.1704360

**Published:** 2026-05-25

**Authors:** Isaie Sibomana, Beth A. Beidleman, J. Philip Karl, Peter S. Figueiredo, Steven D. Landspurg, Janet E. Staab, Camilla A. Mauzy

**Affiliations:** 1 Air Force Research Laboratory, 711 Human Performance Wing, Air and Space Biosciences Division, Wright-Patterson Air Force Base, OH, United States; 2 BlueHalo, an AV Company, Wright-Patterson Air Force Base, OH, United States; 3 Military Nutrition Division, US Army Research Institute of Environmental Medicine, Natick, MA, United States; 4 Oak Ridge Institute of Science and Education, Oak Ridge, TN, United States

**Keywords:** acute mountain sickness, altitude, hypoxia, biomarker, metabolomics, metabolite, risk, susceptibility

## Abstract

Rapid ascent to high altitude (HA) in unacclimatized lowlanders elicits a series of hematologic, ventilatory, metabolic, and cardiovascular adaptations to counteract the lower partial pressure of oxygen. When ascent occurs faster than the body can acclimatize, high-altitude illnesses (HAIs) can occur. The most prevalent of the HAIs is acute mountain sickness (AMS), which can range in severity from mild (minor inconvenience) to severe (total incapacitation). Symptoms of AMS include headache, fatigue, gastrointestinal distress, dizziness, and in some cases sleep disturbances. Identifying individuals at risk for severe AMS, prior to their ascent to HA, would be useful to initiate appropriate prophylaxis approaches in those individuals prior to ascent. We previously reported eight urinary metabolites, measured prior to HA exposure, that discriminated individuals susceptible to moderate or severe AMS from those resistant to AMS (NoAMS). These metabolites include creatine, acetylcarnitine, 3-methylhistidine, N-methylhistidine, hypoxanthine, 1-methylnicotinamide, taurine, and 4-hydroxyphenylpyruvate. This follow-on study examined 41 unacclimatized, physically active healthy soldiers (mean ± SD; age=26 ± 5yr) who were tested at their baseline residence (BLR, 331 m), transported to Taos, NM (2845 m), then immediately hiked (active ascent; n=21) or were driven (passive ascent; n=20) to a HA (3600 m) facility where they resided for four days. AMS was assessed using the shortened version of the Environmental Symptoms Questionnaire (ESQ) and AMS-C scores were calculated. Participants were categorized into resistant (NoAMS; AMS-C< 0.7), mild AMS (mAMS; AMS-C ≥0.7 but <1.53), and moderate to severe AMS (sAMS; AMS-C ≥1.53) groups. Urine samples collected at BLR and HA were analyzed using proton nuclear magnetic resonance (NMR) spectroscopy. BLR urinary metabolite profiles were significantly different (*p ≤* 0.05) between sAMS vs. NoAMS individuals, identifying an AMS risk pattern *prior* to HA exposure. Differentially expressed metabolites in sAMS group included elevated levels of creatine, acetylcarnitine, 3-methylhistidine, isobutyrate, and decreased levels of N-methylhistidine, hypoxanthine, taurine and 1-methylnicotinamide. Interestingly, most of the metabolites that distinguish the different AMS groups are linked to energy production, corroborating findings from our previous study. As urinary levels of these metabolites directly or indirectly reflect the status of the metabolic pathways involved in energy production, these pathways can potentially influence physiologic outcomes to hypoxia.

## 1 Introduction

Exposure to high altitude (HA) subjects the human body to a lower barometric pressure and resultant lower partial pressure of oxygen. Generally, high altitude is defined as an elevation higher than 1500 meters above sea level (masl) (Baniya et al., 2017). However, elevated localities can be further classified as high, very high, and extreme altitudes reflecting elevations of 1500 – 3500 masl, 3500-5500 masl, and 5500-8850 masl, respectively (Savioli et al., 2022). A rapid ascent of unacclimatized individuals to ≥ 2500 masl is associated with a risk of developing acute mountain sickness (AMS). Approximately 25% of visitors to moderate altitudes develop AMS symptoms (Savioli et al., 2022). Above 4000 masl, the risk of AMS ranges from 60% to 90% of individuals (Beidleman et al., 2013) which presents a significant problem for mountaineers, athletes, soldiers, and occupational workers ascending to high altitude.

Although pre-acclimatization can offer protection against developing high altitude illness (HAI), sickness can still occur if the ascent is performed at an extreme altitudes or the ascent is too fast for acclimatization processes to occur (Masuet-Aumatell et al., 2017; Honigman et al., 1993; Roach et al., 1995; Hackett and Roach, 2001; Gallagher and Hackett, 2004; Richalet and Lhuissier, 2015). Exposure to hypoxic conditions triggers a series of physiological responses activating several cellular mechanisms to (1) increase blood flow (by dilatating skeletal muscle vasculature) (Dinenno, 2016), (2) upregulate erythropoietin expression to increase hemoglobin levels (Sawka et al., 2000), (3) increase cellular oxygen delivery (Bartsch and Gibbs, 2007) and (4) downregulate oxygen demand and metabolic rate (Gu and Jun, 2018). Changes in these processes may trigger AMS, as characterized by a variety of symptoms such as headache, fatigue, nausea, vomiting, anorexia, dizziness and in some cases poor sleep (Hackett and Oelz, 1992; Hackett and Roach, 2001; Barry and Pollard, 2003; Roach et al., 2018; Richalet et al., 2021). AMS symptoms usually develop within 6-12 h after a rapid ascent to a high altitude (HA) environment (Honigman et al., 1993; Bartsch and Swenson, 2013; Beidleman et al, 2013) and often subside after 24–36 h if no further increase in elevation occurs (Savioli et al., 2022). Conversely, if the ascent progresses despite the onset of AMS symptoms, the sickness may turn into life-threatening forms of altitude-induced illness such as high altitude cerebral edema (HACE) and high altitude pulmonary edema (HAPE) (Gallagher and Hackett, 2004; Bhagi et al., 2014; Baniya et al., 2017; Luks et al., 2017; Berger and Luks, 2023). Notably, AMS risk varies with each individual as well as elevation of the ascent, and outcome can be tied to environmental, behavioral, and intrinsic risk factors (Hultgren and Marticorena, 1978; Roach et al., 1995; Hultgren et al., 1996; Hackett and Roach, 2001). HA illnesses are rare among Sherpas or other individuals of Tibetan origin living in Nepal or India due to their natural adaptation to high altitude environments. Populations that inhabited highland environments over several generations have been subjected to selective pressures and have acquired genetic adaptations to hypoxic conditions (Witt and Huerta-Sánchez, 2019) making them less likely to develop HAI.

AMS level and severity can be identified by evaluation of symptoms using the shortened version of the Environmental Symptoms Questionnaire (ESQ) (Beidleman et al., 2007) or the Lake Louise 2018 Questionnaire (Roach et al., 2018). Scores generated using the ESQ can be used to classify mild, moderate and severe outcomes at high altitude (Beidleman et al., 2013). This classification has been further used to examine differences in metabolic pathway by-products that can serve as unique biological identifiers of these AMS categories prior to altitude exposure (Sibomana et al., 2021). AMS-susceptible individuals do not display uniform symptoms or symptom severity. Despite similar hypoxic conditions, some individuals develop mild illness while others become seriously ill. This finding suggests the existence of genetic features that modulate AMS symptoms which drive the range of AMS severity. These features may be reflected in measurable metabolites contained in biological specimens such as blood, saliva, and urine. Thus, specific metabolites may serve as potential biomarker classifiers of three categories of AMS status consisting of (1) no AMS (AMS resistant), (2) mild AMS, and (3) moderate to severe AMS. These biomarker classifiers can serve as predictors for AMS risk and potentially predictive of outcome severity. This information is crucial for the development or implementation of targeted individual strategies which attenuate AMS with minimal to no side effects.

Our previous investigations reported eight urinary metabolites that were found discriminating between AMS susceptible from AMS resistant (NoAMS) individuals prior to altitude exposure (Sibomana et al., 2021). These metabolites included creatine, acetylcarnitine, 3-methylhistidine, N-methylhistidine, hypoxanthine, 1-methylnicotinamide, taurine, and 4-hydroxyphenylpyruvate. This current study extended these investigations to include two different factors. The first factor consisted of the active ascent (active ascenders) vs. passive ascent (passive ascenders) to high altitude. The second factor consisted of an addition of a third AMS category, mild AMS, which was previously included in the NoAMS category due to limited sample size and has not been previously examined as a separate grouping. The investigation assessed NoAMS, mild, and severe AMS responses to acute hypoxia and differences in acclimatization timeline between the different groups. This exploratory effort used an untargeted metabolomics approach to identify urinary metabolites that may serve as predictors of AMS susceptibility prior to altitude exposure. The effort also examines how additional energy expenditure incurred during the active ascent to high altitude may influence the AMS prevalence, rate and the acclimatization timeline. Moreover, it provides an insight into the metabolic pathways sustaining susceptibility and resistance to AMS.

## 2 Materials and methods

### 2.1 Subjects and study design

Details on the study design and participants have been previously described in detail (Beidleman et al., 2023). The study was approved by the Headquarters, U.S. Military Research and Development Command Institutional Review Board in Fort Dietrick, MD. Investigators adhered to the policies for the protection of human participants as prescribed by Army Regulation 70-25, and the research was conducted in adherence with the provisions of 32 CFR Part 219.

**Figure 1:**
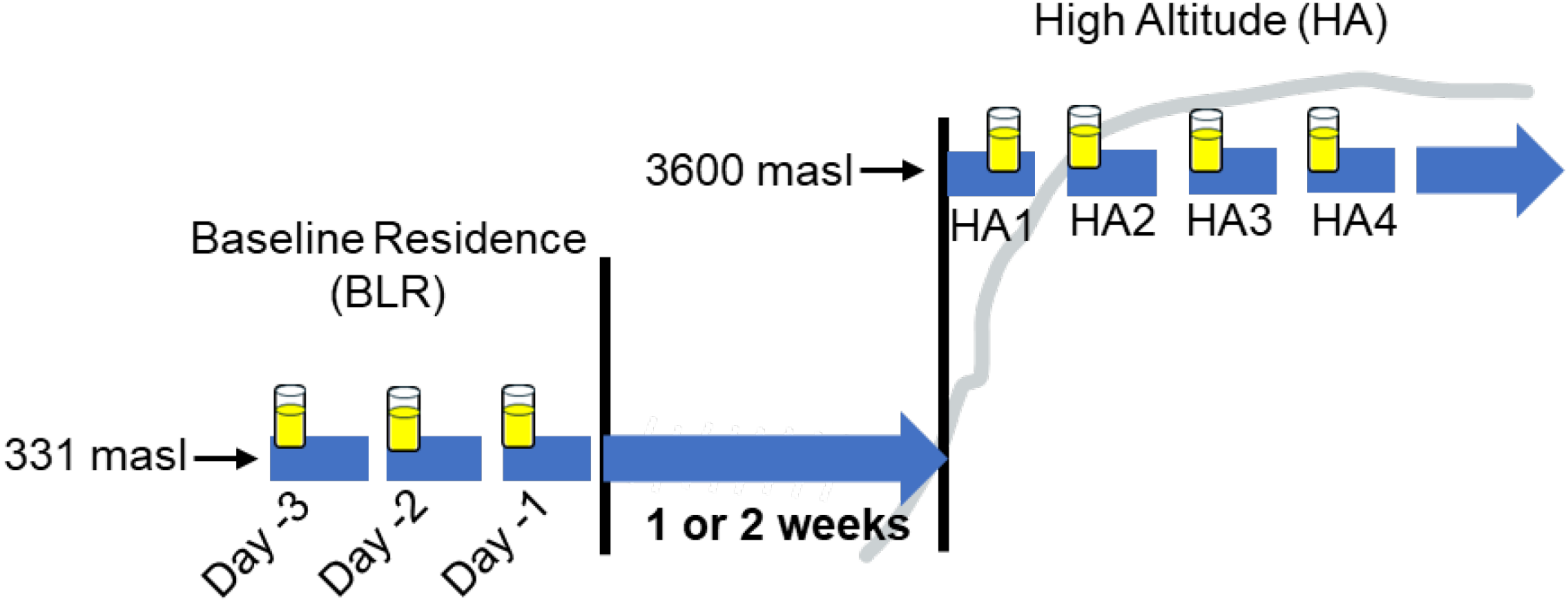
Timeline for urine collection. BLR, baseline level; day -3, first day of urine collection at sea level; day -2, second day of urine collection at sea level; day -1, third day of urine collection at sea level; HA, high altitude; HA1, first day at high altitude, HA2, second day at high altitude; HA3, third day at high altitude; HA4, fourth day at high altitude; masl, meter above sea level.

Forty-one healthy, unacclimatized, physically active subjects 19-38 years of age (mean ± SD; age=26 ± 5yr) participated in the study. Although the study enrollment was open to both sexes, only two women participated. Briefly, the human study was a randomized, controlled trial consisting of baseline residence (BLR) and high altitude testing phases (**Figure 1**). During the first phase, participants were tested for 3 days at their baseline residence (Ft. Leonard Wood, MO; 331 masl). During BLR testing, they consumed a self-selected diet, maintained habitual exercise routines, and were not confined or regulated. After 1-2 weeks (this varied based on which study iteration participants were assigned), participants were transported to Taos, NM (2845 masl), then immediately hiked (active ascent; n=21) or were driven (passive ascent; n=20) to a high altitude (HA, 3600 masl) facility where they resided for the next four days. The ascent to HA occurred in the afternoon on day 1 and the active ascent cohort was outfitted with a backpack weighing ~15% of their body weight. The passive ascent group started their 30 min vehicle ascent journey approximately 30 min after the active group began their hike. To reach the Ski patrol facility located at 3600 masl, the active ascent cohort ascended 750 masl in elevation on a gravel road with an average grade of 15%. Both groups arrived at Ski patrol facility within 60 min of each other to limit acclimatization effects. The average time for the uphill hiking was 120± 24 min with a range of 60–162 min. During the stay at HA, subjects were given a limited variety of foods from which to choose but could eat from those items *ad libitum*. On days 2 and 3 at HA, all subjects participated in a 1-2 h hiking exercise but were otherwise mostly sedentary and engaged in study or leisure activities. No study measurements were made during the group hikes and were used only to prevent boredom.

During the sojourn at HA, the prevalence and severity of AMS was assessed using the shortened version of the Environmental Symptoms Questionnaire (ESQ) (Beidleman et al., 2007). The ESQ was administered two times on both days 2 and 3 at BLR. At high altitude (HA), the ESQ was administered twice on the first day at HA (HA1), five times on the second and third days at HA (HA2 and HA3, respectively) and once on the fourth day at HA (HA4). The ESQ was used to calculate the AMS-cerebral factor (AMS-C) scores and used to categorize AMS severity ranked as no AMS (NoAMS; AMS-C< 0.7), mild AMS (mAMS; AMS-C ≥ 0.7 and < 1.53), and moderate/severe AMS (sAMS; AMS-C≥ 1.53) (Beidleman et al., 2007).

### 2.2 Urine sample collection and preparation

Urine samples obtained on three consecutive days (day -3, day -2, and day -1) at baseline residence (BLR) one to two weeks prior to HA accent (first morning void) and at HA on day 1 (HA1), day 2 (HA2), day 3 (HA3) and day 4 (HA4) were used for the analyses. On the first day at HA (HA1), samples were collected in the afternoon after both active and passive ascent groups arrived at the Ski patrol facility. From HA2 to HA4, samples were collected as first morning voids. Urine samples collected were frozen on site, then shipped on dry ice to Wright-Patterson Air Force Base (WPAFB), OH, where they were stored at -80 °C until delivered to Ohio State University for proton (^1^H) nuclear magnetic resonance (NMR) analyses. The ^1^H NMR analyses were conducted using an 850 MHz high resolution Bruker NMR spectrometer (Model number, Company, City, State). The preparation of urine samples for ^1^H NMR spectral data acquisition followed the procedure described in Sibomana et al (Sibomana et al., 2017).

### 2.3 NMR data acquisition and processing

All urine samples were processed as described in Sibomana et al (Sibomana et al., 2017). Briefly, frozen urine samples were thawed at 4 °C overnight. A 600 µl aliquot of urine was then mixed with 300 µl of phosphate buffer (0.2 M mono- and disodium phosphate; pH = 7.4) and centrifuged at 13,000 rpm (15,680×g) for 10 min to remove any precipitates. A 550 µl aliquot of the super-natant was transferred to a 5 mm NMR tube and mixed with 150 µl of 2,2′,3,3′-tetradeutero-trimethylsilylpropionic acid (TSP) in deuterium oxide (D_2_O), adjusted to yield a final concentration of 2 mM. TSP served as a chemical shift reference (δ = 0.00 ppm) with D_2_O providing a field-frequency lock for NMR data acquisition. The ^1^H NMR spectra were acquired using high resolution Bruker NMR spectrometer instruments operating at 850 MHz and a probe temperature of 25 °C. NMR spectral data processing and analyses were conducted at WPAFB, using Topspin (Bruker, MA, USA) and Mnova (Mestrelab Research, A Coruña, Spain) software packages.

### 2.4 NMR data analyses

Multivariate data analyses were conducted on binned and scaled spectral data. Binned NMR data were scaled to the entire dataset as reference. Principal Component Analysis (PCA) was used as an unsupervised analysis technique to provide a first approach for data visualization (Jolliffe, 1986; Mahle et al., 2011). OPLS-DA was used to isolate the NMR spectral regions identified as important in segregating the AMS groups.

### 2.5 Quantification of metabolite resonances

NMR spectral regions were compared between AMS groups (NoAMS, mAMS and sAMS) at each time point at SL (day-3, day -2, day -1) and HA (HA1, HA2, HA3, and HA4). Signal intensities were integrated using Topspin software. Specific metabolite resonances were quantified using their measured intensities and intensities of TSP with known concentrations. NMR specific resonances were assigned to metabolites with the aid of literature and on-line databases (HMDB, http://www.hmdb.ca/, www.bmrb.wisc.edu, etc.). Signal intensities were integrated to obtain measurements of metabolite concentrations at each time point.

### 2.6 Creatine, acetylcarnitine, and 3-methylhistidine assays

Creatine, 3-methylhistidine, and acetylcarnitine (ALCAR) assays were performed on additional archived urine samples collected at the same time as samples used for the NMR analysis. These assays were conducted as a secondary method for validation of results obtained with ^1^H NMR. Creatine assays were conducted using an Abcam creatine assay (Abcam ab65339; Cambridge, UK) according to manufacturer instructions. The measurements for urinary 3-methylhistidine were first conducted using ELISA kits purchased from Abbexa (3-MH ELISA kit, Abbexa, USA). To validate data obtained with Abbexa kit, another ELISA kit for 3-methylhistidine was purchased from Mybiosource (3MH ELISA kit, Mybiosource, San Diego, CA). Acetylcarnitine assays were conducted using an ELISA kit (ALCAR ELISA kit, Mybiosource, San Diego, CA).

### 2.7 Statistical analyses

The enrollment of the study participants was open to both sexes. However, only two females participated. For statistical analyses, data for these females were combined with data for males because the urinary concentration of measured metabolites were in the concentration ranges noted for males. A repeated measures ANOVA was conducted to examine effects of hypoxia exposure timeline (SL, HA1, HA2, HA3, and HA4), ascent condition (e.g., active vs. passive ascent), and AMS status (noAMS, mAMS, and sAMS) and their interactions on urine metabolite profiles. The clearance assessment of the mAMS and sAMS groups were examined relative to that of the NoAMS group. For metabolites demonstrating time-by-AMS and active vs. passive ascent interactions (*p* < 0.05), Levine’s and Welch’s tests were conducted to assess the equality of variances between the data for SL, HA1, HA2, HA3, and HA4 or sAMS *vs.* NoAMS, mAMS *vs.* NoAMS, and sAMS *vs.* mAMS groups for each metabolite using the statistical software package JMP^®^ 11.0.0 (SAS Institute, Cary, NC, USA). If Levine’s test was significant (*p* ≤ 0.05), then Welch’s test was used to determine if there were significant differences in the mean values between groups for the metabolite of interest. If Levine’s test was not significant, significance was tested using a one-way ANOVA (*t*-test). If both Levine’s and Welch’s tests were significant (*p* ≤ 0.05), a pairwise Welch test was performed for all pairs of groups.

Results shown in figures are normalized to creatinine and are expressed as mean ± standard error of the mean (S.E.) and considered statistically significant at *p*≤ 0.05. Cohen’s *d* (effect size) (Cohen, 1988) was used as a measure of the magnitude of changes in the level of each urinary metabolite measured within each AMS group at HA relative to BLR by subtracting the value obtained for each group at HA from that obtained at BLR and assessing the difference relative to the within-group pooled standard deviations for HA and BLR (see **Tables 1**, **2**). Effect size was also used as a measure of the magnitude of changes in the level of each urinary metabolite measured at BLR (see **Table 3**) and HA (see **Tables 4**, **5**) for sAMS and mAMS groups relative to NoAMS. This was accomplished by subtracting the value obtained for NoAMS from those obtained for sAMS or mAMS and assessing the difference relative to the pooled standard deviations for NoAMS and sAMS or mAMS.

**Table 1:** Heatmap showing the magnitude of changes (effect sizes or Cohen’s *d*; (Cohen, 1988)) in urinary metabolite clearance measured within each AMS group at HA relative to BLR (sea level) by subtracting the value obtained for each group at HA from that obtained at BLR and assessing the difference relative to the within-group pooled standard deviations for HA and BLR. 
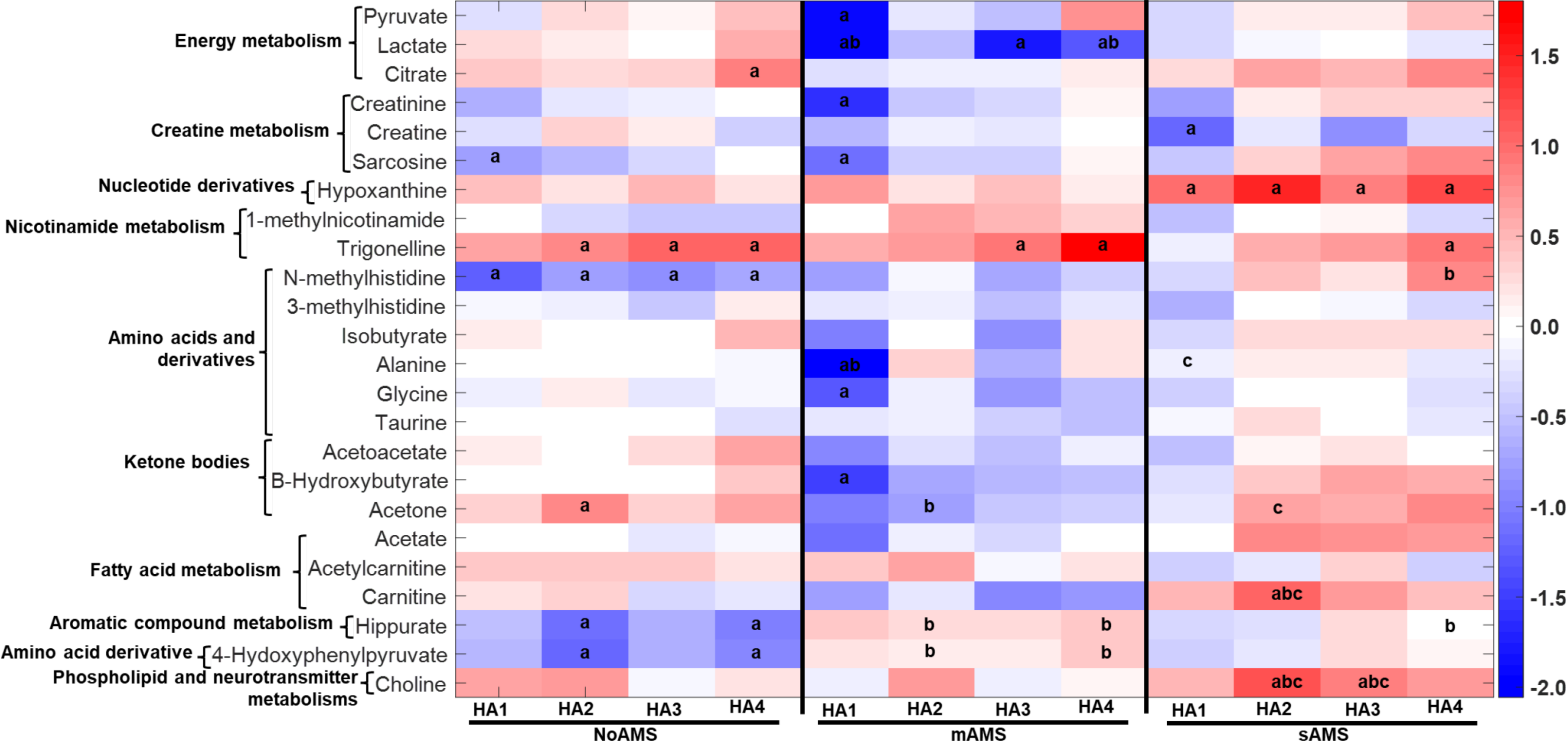

All the subjects were driven (passive ascenders) to Kachina Peak (~3600 masl). The blue color represents a negative effect size (a decrease relative to BLR) while a red color indicates a positive effect size (an increase relative to BLR). Since the statistical analyses were conducted on raw data, the statistical annotations shown for comparisons refer to these data. The letter “a” indicates that HA differs from BLR, letter “b” denotes that NoAMS differs from mAMS and sAMS groups while the letter “c” indicates significant differences between mAMS and sAMS groups (*p* ≤ 0.05). AMS, acute mountain sickness; BLR, baseline residence (sea level); HA, high altitude; HA1, first day at HA; HA2, second day at HA; HA3, third day at HA; HA4, fourth day at HA; NoAMS, no acute mountain sickness; mAMS, mild acute mountain sickness; sAMS, severe acute mountain sickness.

**Table 2:** Heatmap showing the magnitude of changes (effect sizes or Cohen’s *d*; (Cohen, 1988)) in urinary metabolite clearance measured within each AMS group at HA relative to BLR (sea level) by subtracting the value obtained for each group at HA from that obtained at BLR and assessing the difference relative to the within-group pooled standard deviations for HA and BLR. 
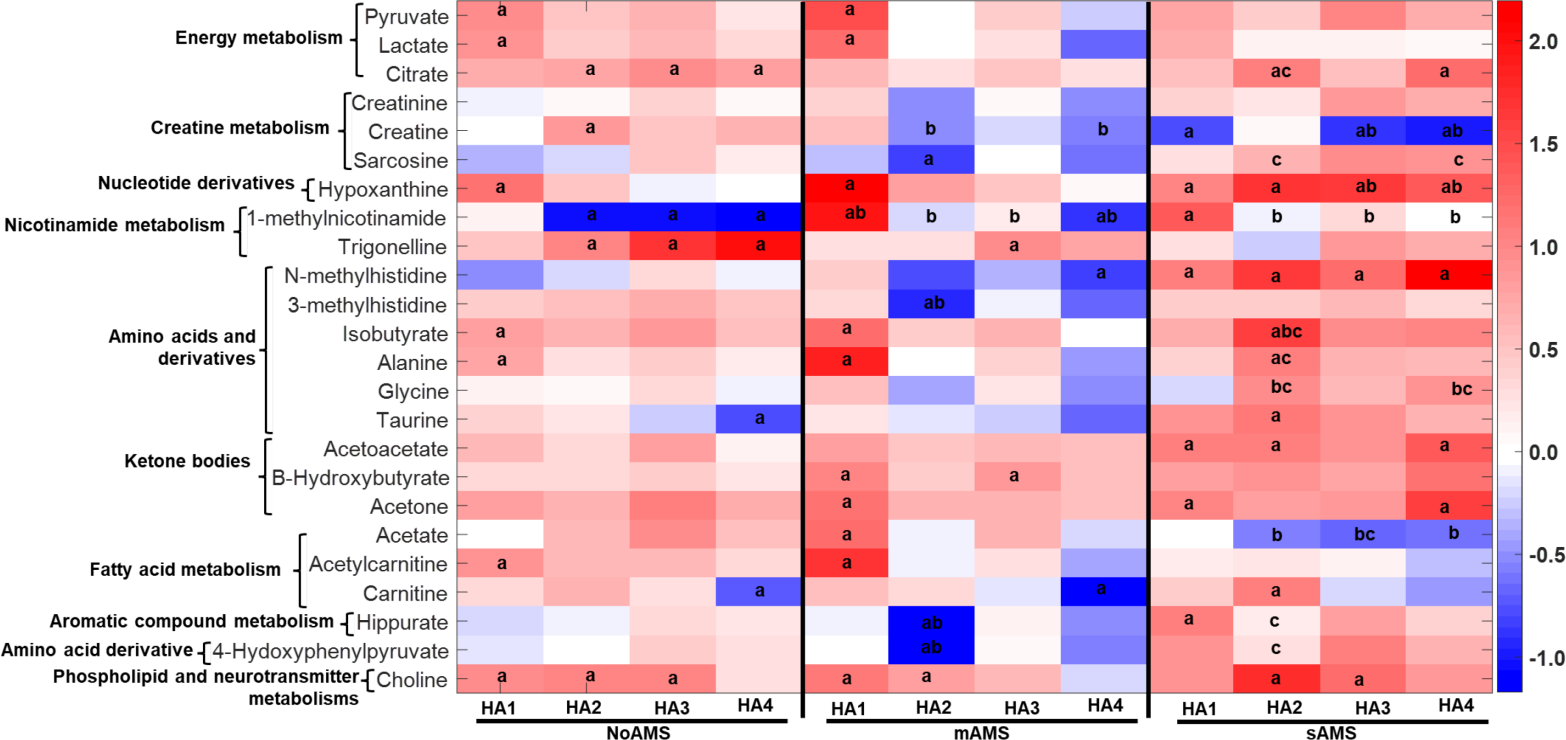

All the subjects hiked (active ascenders) to reach Kachina Peak (~3600 masl). The blue color represents a negative effect size (a decrease relative to BLR) while a red color indicates a positive effect size (an increase relative to BLR). Since the statistical analyses were conducted on raw data, the statistical annotations shown for comparisons refer to these data. The letter “a” indicates that HA differs from BLR, letter “b” denotes that NoAMS differs from mAMS and sAMS groups while the letter “c” indicates significant differences between mAMS and sAMS groups (*p* ≤ 0.05). AMS, acute mountain sickness; BLR, baseline residence (sea level); HA, high altitude; HA1, first day at HA; HA2, second day at HA; HA3, third day at HA; HA4, fourth day at HA; NoAMS, no acute mountain sickness; mAMS, mild acute mountain sickness; sAMS, slevere acute mountain sickness.

**Table 3:** Heatmap showing the magnitude of changes (effect sizes or Cohen’s *d*; (Cohen, 1988)) in urinary metabolites levels at BLR (sea level) (averaged data for three consecutive days) for mAMS and sAMS groups relative to NoAMS group. 
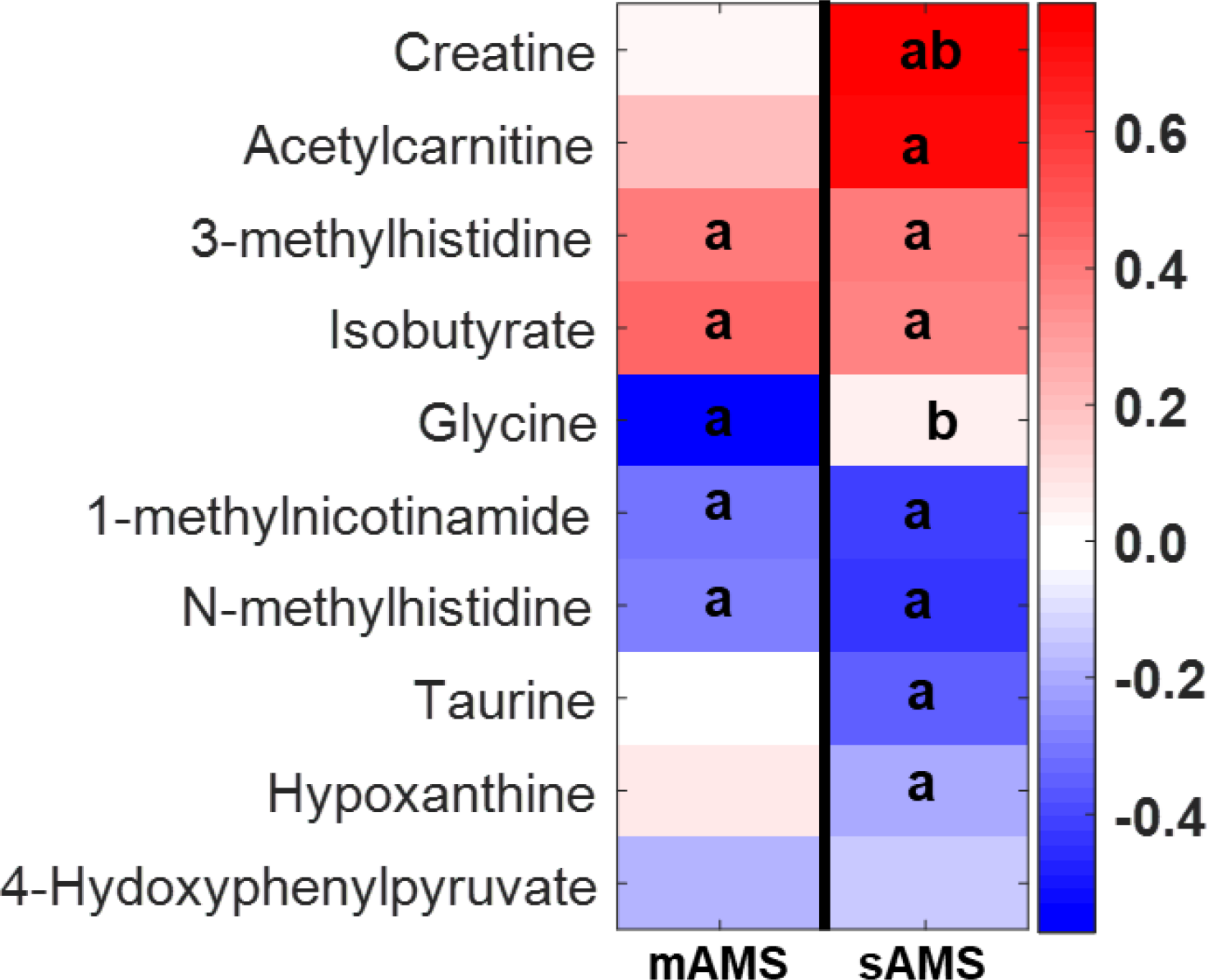

The blue color represents a negative effect size (a decrease relative to NoAMS) while a red color indicates a positive effect size (an increase relative to NoAMS). Heatmap was generated using NMR spectral data for urine samples collected on 3 consecutive days at BLR. Since the statistical analyses were conducted on raw data, the statistical annotations shown for comparisons refer to these data. The letter “a” indicates that NoAMS differs from mAMS and sAMS groups while the letter “b” indicates significant differences between mAMS and sAMS groups (*p* ≤ 0.05). BLR, baseline residence; NoAMS, no acute mountain sickness; mAMS, mild acute mountain sickness; sAMS, severe acute mountain sickness.

**Table 4:** Heatmap showing the magnitude of changes (effect sizes or Cohen’s *d*; (Cohen, 1988)) in urinary metabolite clearance at HA for mAMS and sAMS subjects relative to clearance noted for NoAMS individuals. 
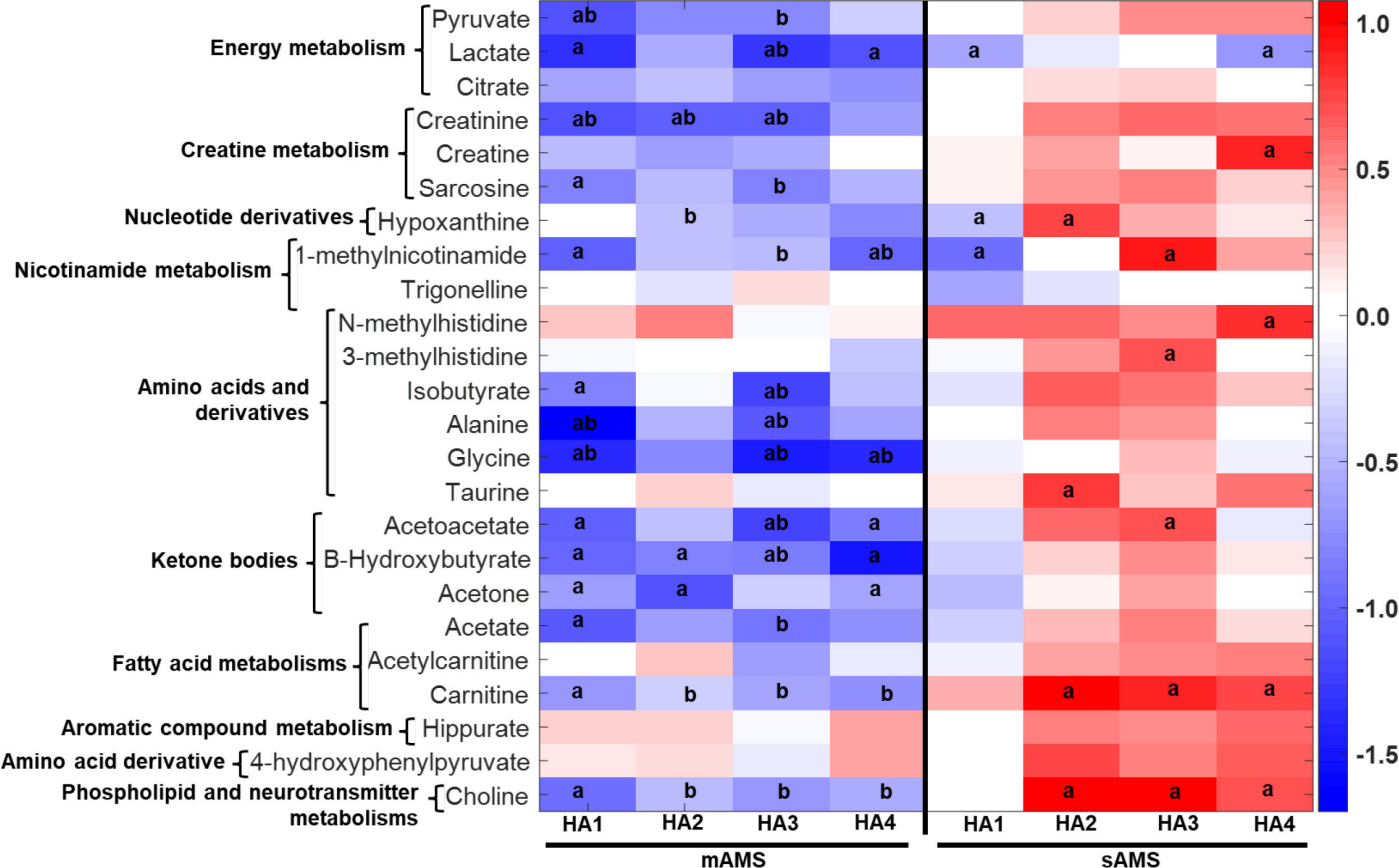

This was accomplished by subtracting the value obtained for NoAMS from that obtained for sAMS or mAMS and assessing the difference relative to the pooled standard deviations for NoAMS and sAMS or mAMS. All the subjects were driven (passive ascenders) to Kachina Peak (~3600 masl). The blue color represents a negative effect size (a decrease relative to NoAMS) while a red color indicates a positive effect size (an increase relative to NoAMS). Since the statistical analyses were conducted on raw data, the statistical annotations shown for comparisons refer to these data. The letter “a” indicates that NoAMS differs from mAMS and sAMS groups while the letter “b” indicates significant differences between mAMS and sAMS groups (p ≤ 0.05). HA, high altitude; HA1, first day at HA; HA2, second day at HA; HA3, third day at HA; HA4, fourth day at HA; NoAMS, no acute mountain sickness; mAMS, mild acute mountain sickness; sAMS, severe acute mountain sickness.

**Table 5:** Heatmap showing the magnitude of changes (effect sizes or Cohen’s *d*; (Cohen, 1988)) in urinary metabolite clearance at HA for mAMS and sAMS subjects relative to clearance noted for NoAMS individuals. 
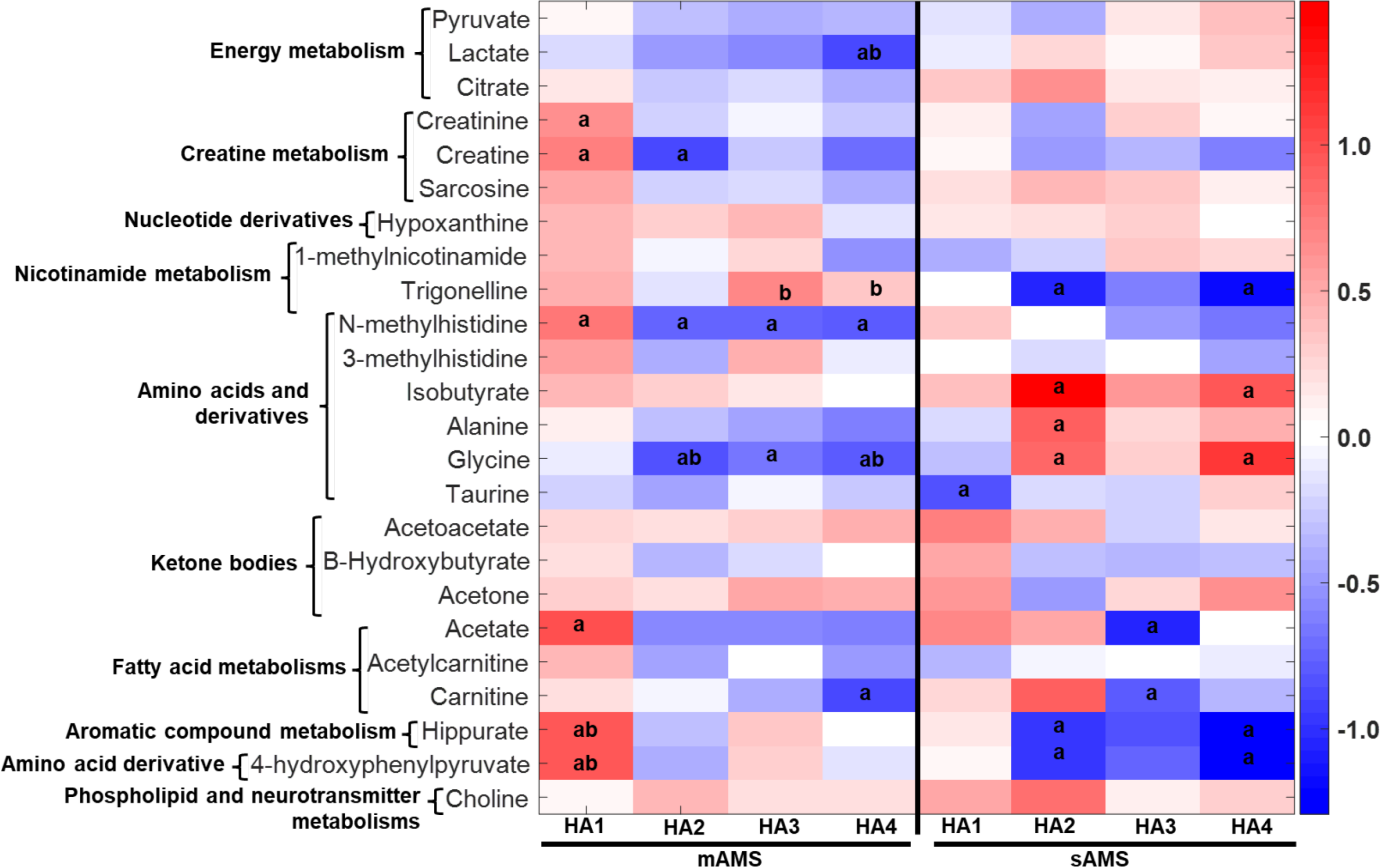

This was accomplished by subtracting the value obtained for NoAMS from that obtained for sAMS or mAMS and assessing the difference relative to the pooled standard deviations for NoAMS and sAMS or mAMS. All the subjects ascended on foot (active ascenders) to Kachina Peak (~3600 masl). The blue color represents a negative effect size (a decrease relative to NoAMS) while a red color indicates a positive effect size (an increase relative to NoAMS). Since the statistical analyses were conducted on raw data, the statistical annotations shown for comparisons refer to these data. The letter “a” indicates that NoAMS differs from mAMS and sAMS groups while the letter “b” indicates significant differences between mAMS and sAMS groups (*p* ≤ 0.05). HA, high altitude; HA1, first day at HA; HA2, second day at HA; HA3, third day at HA; HA4, fourth day at HA; NoAMS, no acute mountain sickness; mAMS, mild acute mountain sickness; sAMS, severe acute mountain sickness.

**Figure 2:**
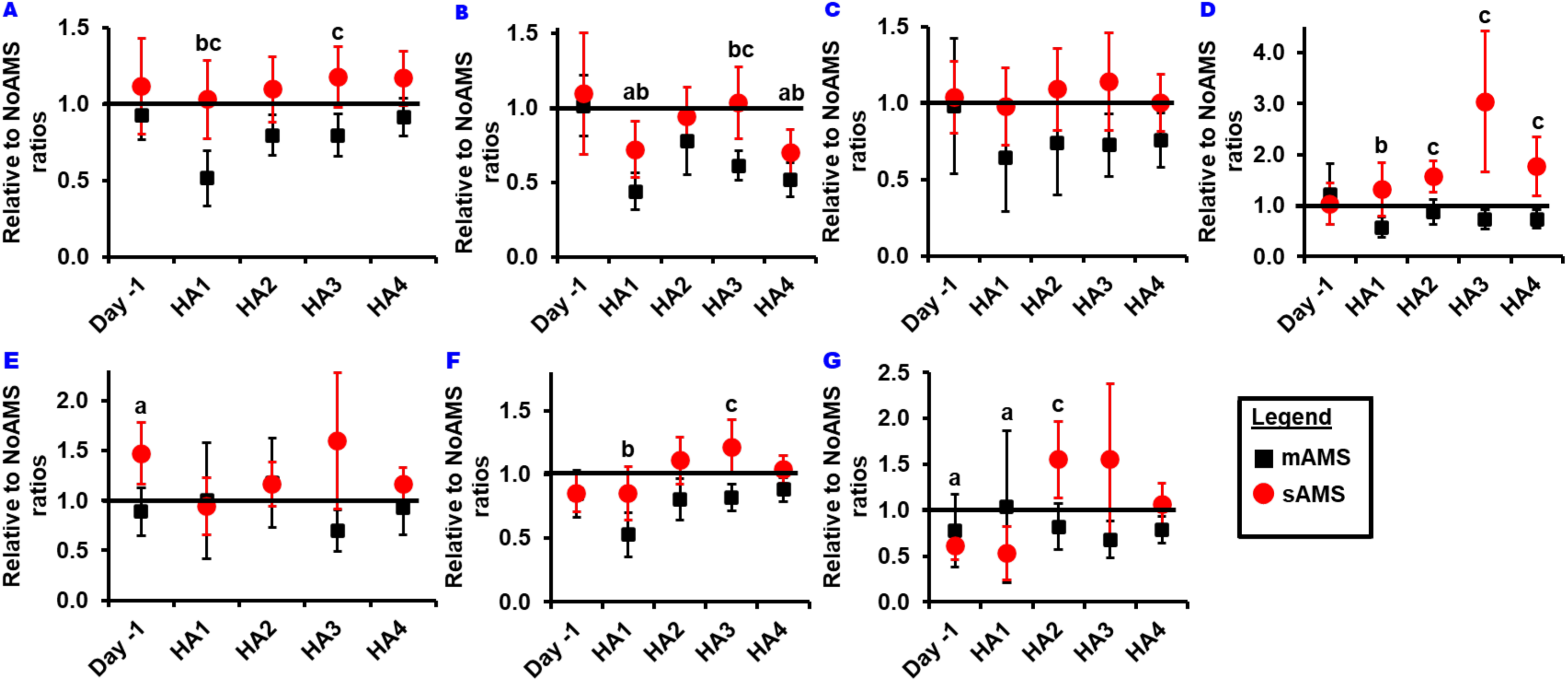
mAMS/NoAMS and sAMS/NoAMS ratios for urinary clearance of metabolites linked to (1) energy metabolism such as **(A)** pyruvate, **(B)** lactate, **(C)** citrate, **(D)** carnitine, **(E)** acetylcarnitine, **(F)** acetate and **(G)** hypoxanthine at SL and high altitude for the subjects who were driven (passive ascenders) to Kachina peak. Letters “a” and “b” indicate that sAMS and mAMS significantly differ from NoAMS, respectively while the letter “c” denotes significant difference between sAMS and mAMS (*p ≤* 0.05). Day -1, third (last) day of urine collection at baseline residence (sea level); HA, high altitude; HA1, first day at HA; HA2, second day at HA; HA3, third day at HA; HA4, fourth day at HA; mAMS, mild acute mountain sickness; NoAMS, no acute mountain sickness; sAMS, severe acute mountain sickness.

**Figure 3:**
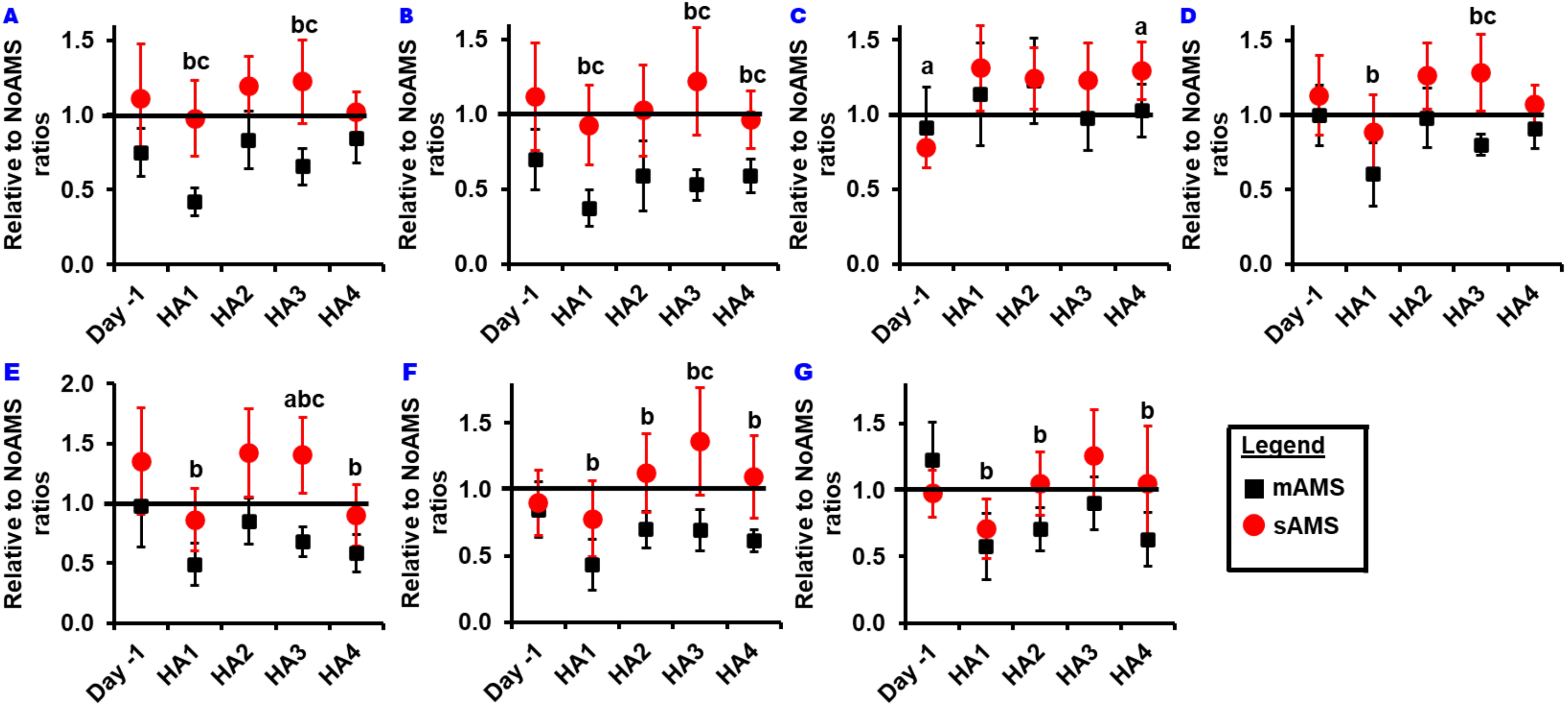
mAMS/NoAMS and sAMS/NoAMS ratios for urinary clearance of (1) amino acids and derivatives such as **(A)** alanine, **(B)** glycine, **(C)** N-methylhistidine, **(D)** isobutyrate, (2) ketone bodies such as **(E)** acetoacetate, **(F)** β-hydroxybutyrate, and **(G)** acetone at SL and high altitude for the subjects who were driven (passive ascenders) to Kachina peak. Letters “a” and “b” indicate that sAMS and mAMS significantly differ from NoAMS, respectively while the letter “c” denotes significant difference between sAMS and mAMS (*p ≤* 0.05). Day -1, third (last) day of urine collection at baseline residence (sea level); HA, high altitude; HA1, first day at HA; HA2, second day at HA; HA3, third day at HA; HA4, fourth day at HA; mAMS, mild acute mountain sickness; NoAMS, no acute mountain sickness; sAMS, severe acute mountain sickness.

**Figure 4:**
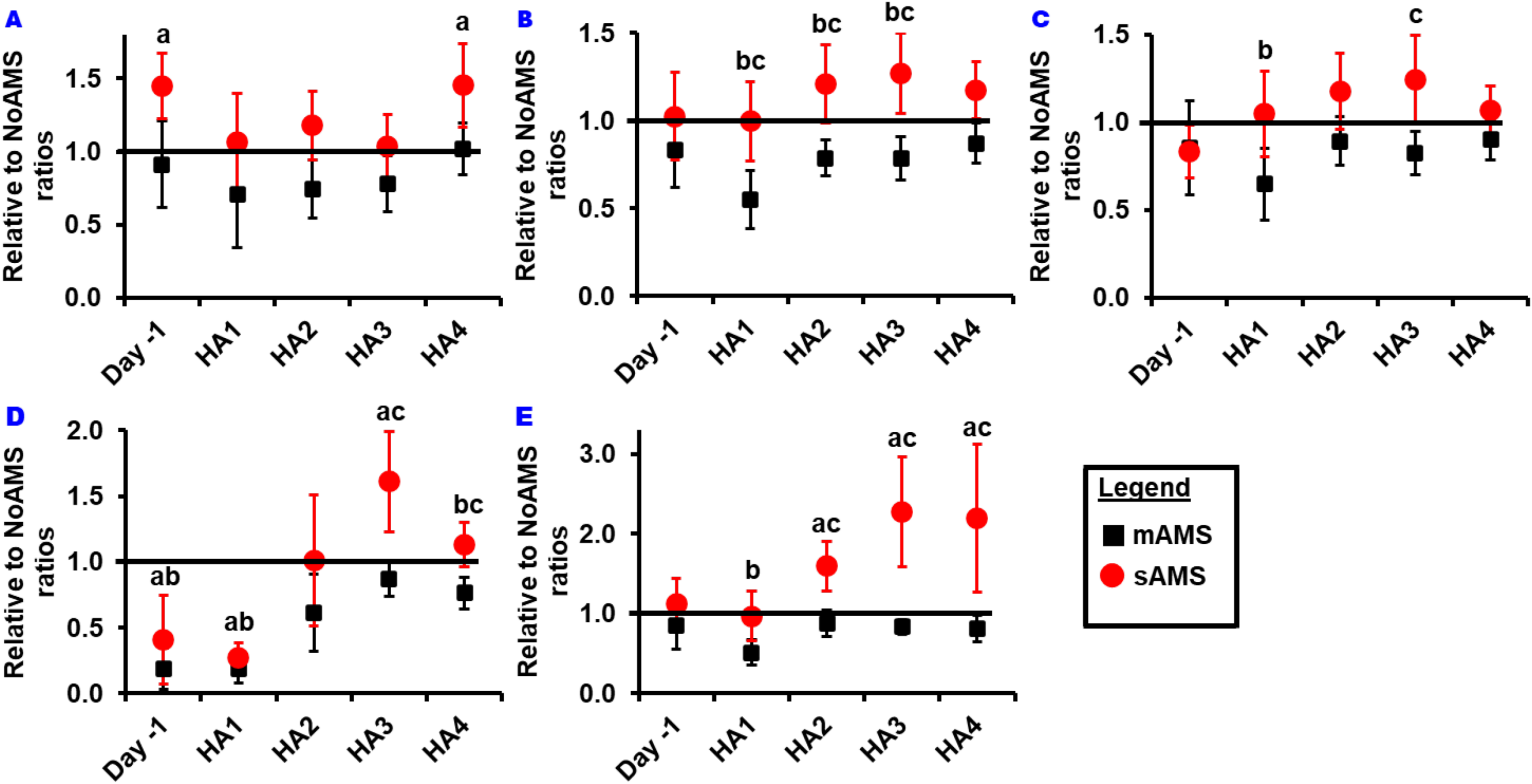
mAMS/NoAMS and sAMS/NoAMS ratios for urinary clearance of metabolites (1) linked to creatine metabolism such as **(A)** creatine, **(B)** creatinine, **(C)** sarcosine and (2) deriving from other pathways such as **(D)** 1-methylnicotinamide, **(E)** choline for mAMS and sAMS groups at SL and high altitude for the subjects who were driven (passive ascenders) to Kachina peak. Letters “a” and “b” indicate that sAMS and mAMS significantly differ from NoAMS, respectively while the letter “c” denotes significant difference between sAMS and mAMS (*p ≤* 0.05). Day -1, third (last) day of urine collection at baseline residence (sea level); HA, high altitude; HA1, first day at HA; HA2, second day at HA; HA3, third day at HA; HA4, fourth day at HA; mAMS, mild acute mountain sickness; NoAMS, no acute mountain sickness; sAMS, severe acute mountain sickness.

**Figure 5:**
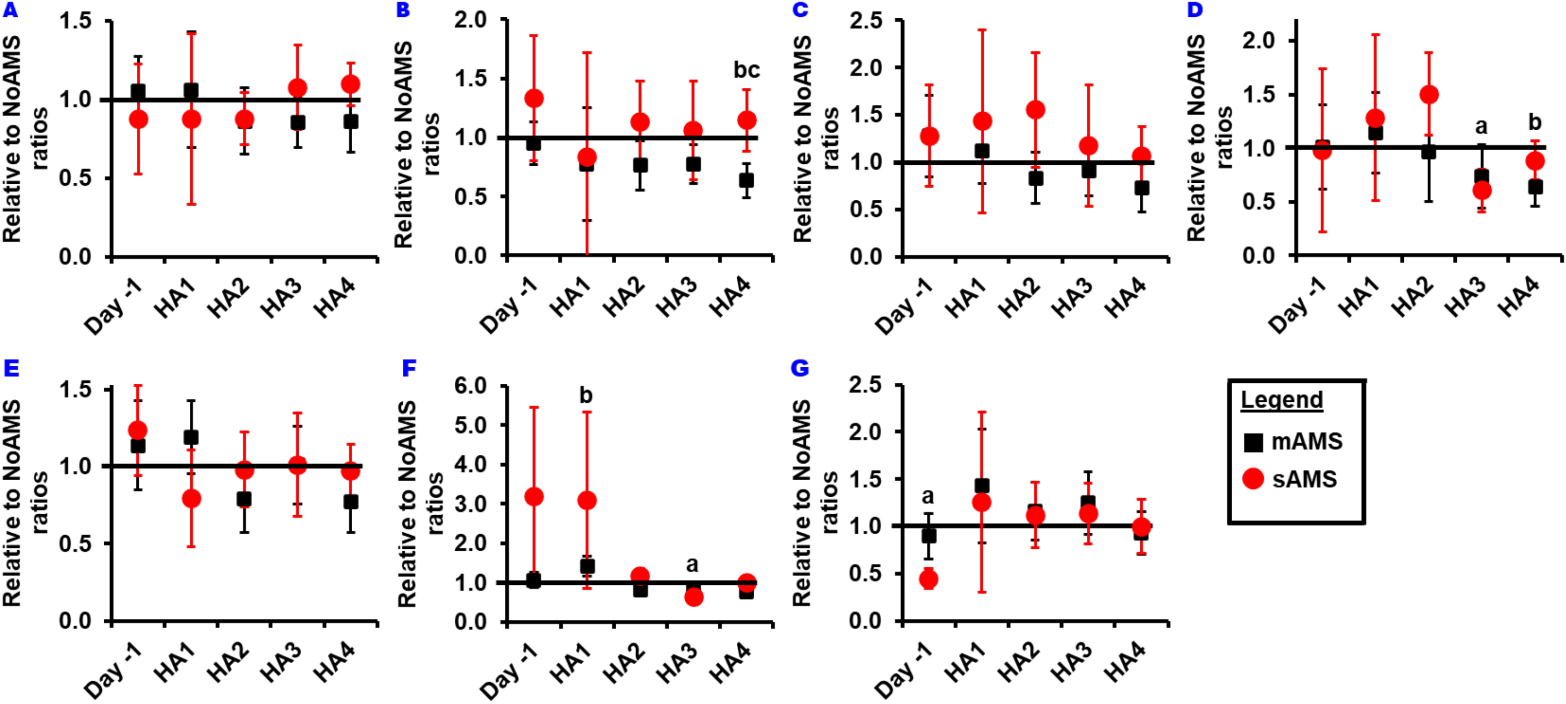
mAMS/NoAMS and sAMS/NoAMS ratios for urinary clearance of metabolites linked to (1) energy metabolism such as **(A)** pyruvate, **(B)** lactate, **(C)** citrate, **(D)** carnitine, **(E)** acetylcarnitine, **(F)** acetate and **(G)** hypoxanthine at SL and high altitude for the subjects who ascended by foot (active ascenders) to reach Kachina peak. Letters “a” and “b” indicate that sAMS and mAMS significantly differ from NoAMS, respectively while the letter “c” denotes significant difference between sAMS and mAMS (*p ≤* 0.05). Day -1, third (last) day of urine collection at baseline residence (sea level); HA, high altitude; HA1, first day at HA; HA2, second day at HA; HA3, third day at HA; HA4, fourth day at HA; mAMS, mild acute mountain sickness; NoAMS, no acute mountain sickness; sAMS, severe acute mountain sickness.

**Figure 6:**
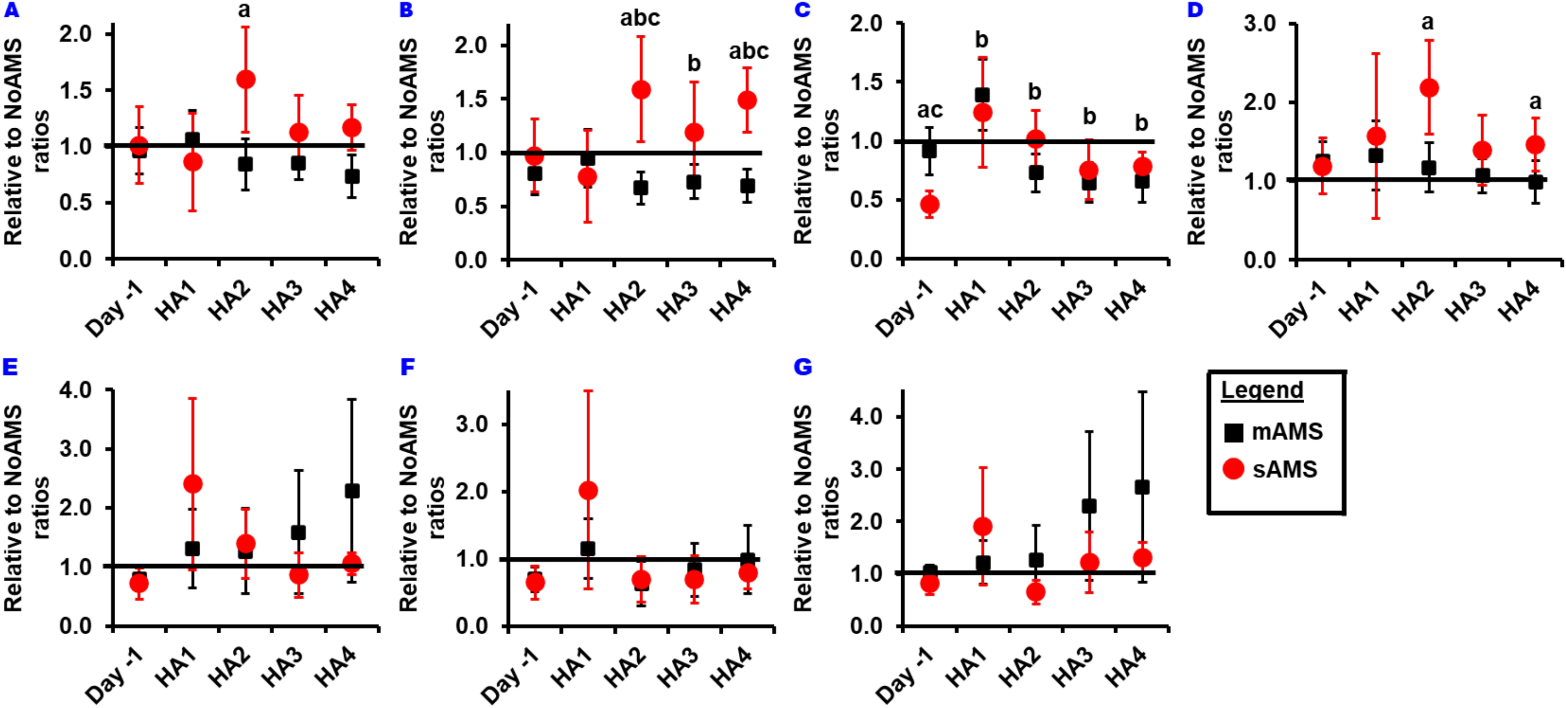
mAMS/NoAMS and sAMS/NoAMS ratios for urinary clearance of (1) amino acids and derivatives such as **(A)** alanine, **(B)** glycine, **(C)** N-methylhistidine, **(D)** isobutyrate, (2) ketone bodies such as **(E)** acetoacetate, **(F)** β-hydroxybutyrate, and **(G)** acetone at SL and high altitude for the subjects who ascended by foot (active ascenders) to reach Kachina peak. Letters “a” and “b” indicate that sAMS and mAMS significantly differ from NoAMS, respectively while the letter “c” denotes significant difference between sAMS and mAMS (*p ≤* 0.05). Day -1, third (last) day of urine collection at baseline residence (sea level); HA, high altitude; HA1, first day at HA; HA2, second day at HA; HA3, third day at HA; HA4, fourth day at HA; mAMS, mild acute mountain sickness; NoAMS, no acute mountain sickness; sAMS, severe acute mountain sickness.

**Figure 7:**
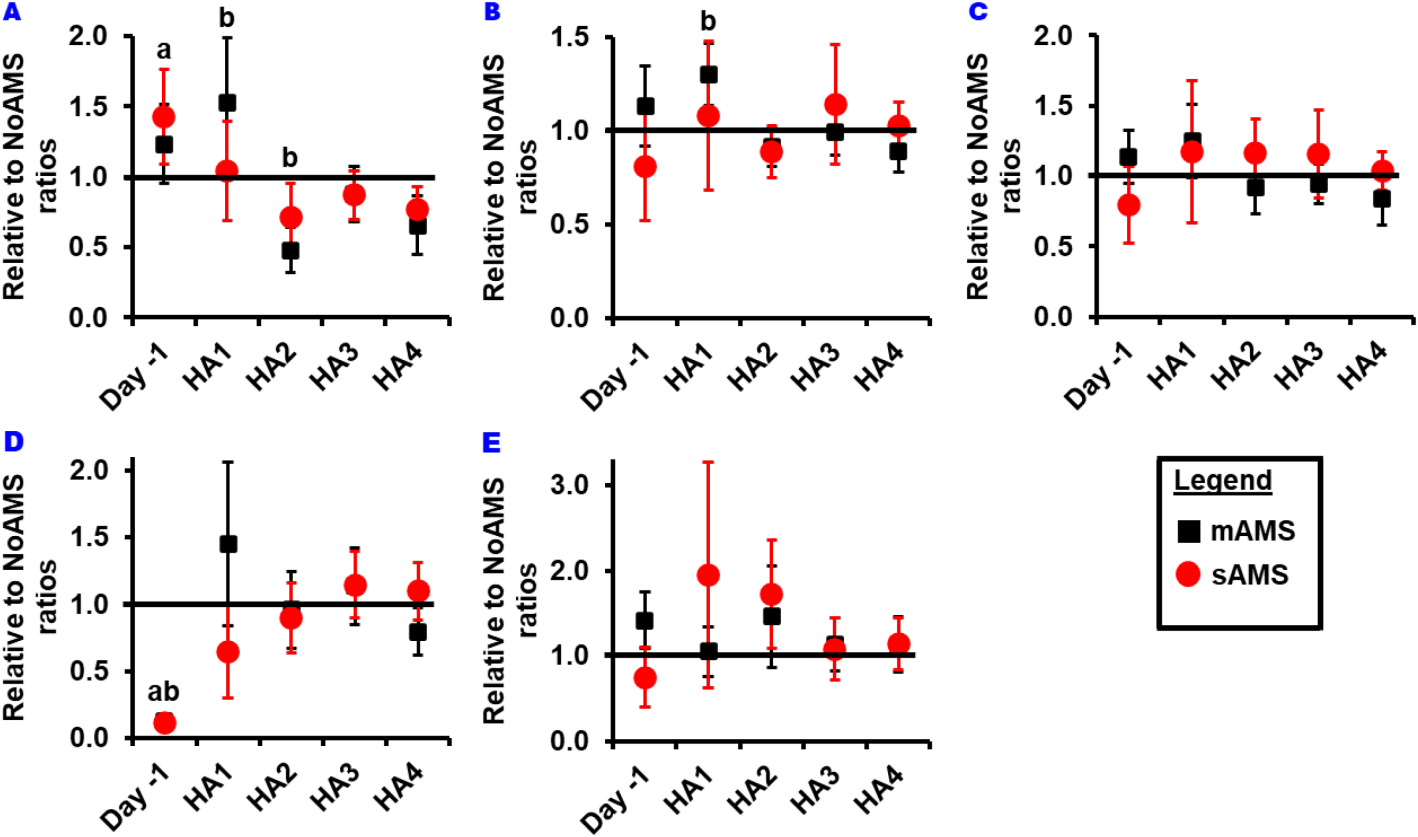
mAMS/NoAMS and sAMS/NoAMS ratios for urinary clearance of metabolites (1) linked to creatine metabolism such as **(A)** creatine, **(B)** creatinine, **(C)** sarcosine and (2) deriving from other pathways such as **(D)** 1-methylnicotinamide, **(E)** choline for mAMS and sAMS groups at SL and high altitude for the subjects who ascended by foot (active ascenders) to reach Kachina peak. Letters “a” and “b” indicate that sAMS and mAMS significantly differ from NoAMS, respectively while the letter “c” denotes significant difference between sAMS and mAMS (*p ≤* 0.05). Day -1, third (last) day of urine collection at baseline residence (sea level); HA, high altitude; HA1, first day at HA; HA2, second day at HA; HA3, third day at HA; HA4, fourth day at HA; mAMS, mild acute mountain sickness; NoAMS, no acute mountain sickness; sAMS, severe acute mountain sickness.

To determine how much the urinary clearance levels of measured metabolites for mAMS and sAMS subjects deviated from the clearance levels noted for NoAMS individuals, mAMS/NoAMS and sAMS/NoAMS ratios were calculated. Values of these calculations indicated the magnitude of metabolite clearance differences between AMS susceptible (mAMS and sAMS) subjects and AMS resistant (NoAMS) individuals. The results for passive ascent cohort are displayed in **Figures 2**–**4** while those for active ascenders are shown in **Figures 5**–**7**.

## 3 Results

### 3.1 Incidence and severity of acute mountain sickness

The incidence of AMS for the current study was 54% (27% for each of mAMS and sAMS groups) (**Table 6**). The overall mean peak AMS-weighted cerebral factor scores (AMS-C) for sAMS (2.64 ± 0.20; n=11; 26.83%) and mAMS (1.01 ± 0.07; n=11; 26.83%) individuals were significantly elevated (*p* < 0.05) compared to the score for NoAMS subjects (0.31 ± 0.03; n=19; 46.34%) (**Table 6**). The AMS score for sAMS group was also significantly higher than the score for mAMS group. Considering pre-altitude exposure energy expenditure differences (i.e., passive ascent vs. active ascent), AMS incidences were also examined to determine if the uphill hiking has a significant impact on AMS occurrence. The peak AMS-C scores in the passive ascenders for sAMS (2.54 ± 0.20; n=7; 35.00%) and mAMS (1.13 ± 0.15; n=4; 20.00%) significantly elevated (p < 0.05) compared to the scores for NoAMS passive ascenders (0.33 ± 0.45; n=9; 45.00%) (**Table 6**). Similar comparisons were conducted using the active ascender data. The AMS-C scores for active ascenders for sAMS (2.82 ± 0.46; n=4; 19.05%) and mAMS (0.95 ± 0.08; n=7; 33.33%) were significantly elevated relative to the scores for NoAMS active ascenders (0.29 ± 0.04; n=10; 47.62%). In general, uphill hiking tended to increase the AMS incidence rate for mAMS group (33.33%) compared to the incidence rate noted for the mAMS subjects belonging to the passive ascent group (20.00%). Conversely, the uphill hike tended to reduce the rate of sAMS compared to the rate observed for the sAMS passive ascenders (19.05% vs. 35.00% for sAMS active ascenders and sAMS passive ascenders, respectively).

**Table 6:** AMS scores for subjects enrolled in the study.

Groups	NoAMS	mAMS	sAMS
Passive and active Ascent groups	0.31 ± 0.03 (n=19; 46.34%)	1.01 ± 0.07 (n=11; 26.83%)	2.64 ± 0.20^ab^ (n=11; 26.83%)
Passive Ascent group	0.33 ± 0.45 (n=9; 45.00%)	1.13 ± 0.15^a^ (n=4; 20.00%)	2.54 ± 0.20^ab^ (n=7; 35.00%)
Active Ascent group	0.29 ± 0.04 (n=10; 47.62%)	0.95 ± 0.08^a^ (n=7; 33.33%)	2.82 ± 0.46^ab^ (n=4; 19.05%)

The letter “a” indicates that mAMS and sAMS groups significantly differ from NoAMS group while the letter “b” denotes significant differences between mAMS and sAMS groups (p ≤ 0.05).

In general, the AMS scores for the active ascent group tended to decline faster than those in the passive ascent group, regardless of the AMS status. Most of the subjects (8 out of 11 subjects) who experienced mild or severe AMS in the active ascent group displayed their highest AMS scores on the first day of HA exposure compared to those who were in the passive ascent group (2 out of 11 subjects). A higher number of subjects in the passive ascent group experienced mild or severe AMS on the second day (5 out of 11 subjects) and third day (4 out of 11 subjects) of HA exposure.

### 3.2 Altitude exposure-induced alterations in urinary metabolite clearance over time

Passive Ascent Cohort. To isolate the altitude-induced effects on the urine metabolite profile apart from the exercise effect, data was examined for the passive ascent group only. Based on previous metabolite profile response data (Sibomana et al., 2021), the HA molecular responses were separated into two phases consisting of an acute phase (first 24 hrs at HA) and an acclimatization phase (>24 hr HA). Significant changes in urinary metabolite profiles were noted when the subjects moved from BLR to HA as well as during the entire four-day HA sojourn regardless of AMS status (**Table 1**). The urinary levels of each metabolite measured at HA were compared to its level noted at BLR. Examination of the spectral data for urine samples collected from NoAMS, mAMS, and sAMS passive ascenders indicated alterations in metabolites involved in various cellular metabolic pathways. While most clearance alterations for NoAMS and sAMS passive ascent groups were noted on the second day of HA exposure (HA2), mAMS passive ascenders displayed more pronounced metabolite excretion on the first day of HA exposure (HA1) during the acute response to hypoxia (**Table 1**). During this phase, significant clearance alterations of seven metabolites were noted for mAMS group compared to NoAMS and sAMS groups, that displayed only two alterations. Clearance levels for all these seven metabolites were reduced at HA1 relative to their excretion levels measured at BLR. The cellular mechanisms that were affected for mAMS passive ascent group included energy metabolism (decreases in pyruvate, -51%, and lactate, -50%), creatine metabolism (decreases in creatine, -52% and sarcosine, -48%), amino acids (decreases in alanine, -43% and glycine, -51%), and ketone body (β-hydroxybutyrate, -51%). The two significant changes displayed by NoAMS passive ascenders at HA1 included decreases in urinary levels of sarcosine (-32%) and N-methylhistidine (-46%), a histidine degradation derivative. During the acute response phase to hypoxia (HA1), sAMS passive ascenders had reduced urinary creatine levels by 36% relative to BLR with a 70% increase in hypoxanthine clearance, an AMP/GMP degradation by-product.

During what we consider the acclimatization phase (HA2 and beyond), the levels of urinary trigonelline were consistently elevated for the NoAMS passive ascent group (2.3-fold, 1.6-fold and 1.6-fold increases, at HA2, HA3 and HA4, respectively), while N-methylhistidine clearances remained reduced (28%, 33% and 25% decreases, respectively) (**Table 1**). Other NoAMS group clearance changes included citrate, acetone, hippurate and 4-hydroxyphenylpyruvate. In the mAMS passive ascent group, only lactate and trigonelline clearances were significantly affected, with lactate decreasing on HA3 and HA4 (39% and 30% decreases, respectively) while trigonelline increased (2 and 1.5-fold increases, respectively). In the sAMS passive ascent group, excretion of hypoxanthine was consistently elevated during the entire acclimatization phase (1.9-fold, 2.8-fold, and 91% increases at HA2, HA3, and HA4, respectively). Although this group also displayed increased elimination of trigonelline, carnitine, and choline, these alterations were transient as the clearance increases of trigonelline (102%, increase) and carnitine (83%, increase) were only noted at HA4 and HA2, respectively, while the clearance levels for choline were elevated at HA2 (86%) and HA3 (94%). It is noteworthy that in the sAMS passive ascent group, urinary carnitine was significantly elevated at HA2 whereas choline levels were elevated at both HA2 and HA3 relative to the mAMS passive ascent group.

### 3.3 Uphill hiking coupled with altitude exposure altered urinary metabolite clearances

Active Ascent Group. The magnitude of metabolite changes related to a combination of altitude exposure and uphill hiking exercise was assessed using active ascender data. The active ascent group also displayed significant changes in urinary metabolite profiles at HA compared to day -1 at BLR regardless of AMS status (**Table 2**). While the NoAMS and mAMS passive ascenders responded to hypoxic conditions by reducing metabolite excretion during the acute hypoxic response phase compared to BLR (**Table 1**), the NoAMS, mAMS and sAMS active ascenders increased urinary metabolite elimination during this phase (**Table 2**). More significant metabolite clearance alterations were displayed by the NoAMS and mAMS active ascenders during the acute hypoxia phase compared to any time during the acclimatization phase (HA2, HA3, and HA4). At HA1, NoAMS and mAMS active ascent groups respectively increased the clearance of metabolites (1) involved in energy production (pyruvate, 1.6-fold vs. 1.6-fold and lactate 21.7-fold vs. 17.5-fold), fatty acid metabolism (acetylcarnitine, 70% vs. 78%), and phospholipid/neurotransmitter metabolism (choline, 113% vs 58%) or (2) belonging to categories such as nucleotide derivatives (hypoxanthine, 10.1-fold vs. 16.8-fold), amino acid and derivatives (alanine, 65% vs. 83% and isobutyrate, 95% vs. 107%) compared to clearance noted at SL. Besides these changes, mAMS active ascent group also displayed significantly elevated levels of metabolites linked to nicotinamide metabolism (1-methylnicotinate, 12.3-fold), ketone body metabolism (β-hydroxybutyrate, 118% and acetone, 116%) and fatty acid metabolism (acetate, 33%). During the acute hypoxic response phase, sAMS active ascenders reduced creatine excretion (26% decrease) and increased clearances of six metabolites (hypoxanthine, 1-methylnicotinamide, N-methylhistidine, acetoacetate, acetone, and hippurate). Creatine was the only metabolite that decreased as a response to uphill hiking combined with HA exposure.

The most profound changes during the acclimatization phase were seen in the sAMS active ascent group compared to NoAMS and mAMS active ascent groups (**Table 2**). Examination of metabolite profiles for the sAMS active ascenders indicated that the urinary hypoxanthine and N-methylhistidine were significantly elevated during both the acute hypoxic response (30.2-fold and 5.5-fold increases, respectively) and acclimatization (ranging from 1.2-fold to 2.1-fold and from 63% to 102%, respectively) relative to levels noted at BLR. Clearance increases for citrate, amino acids, and derivatives (alanine, isobutyrate, and taurine), acetoacetate, carnitine, and choline noted for this group were transient. Creatine was the only metabolite for which the clearance was significantly reduced during the acute response (26% decrease) and acclimatization (20% and 25% decreases at HA3 and HA4, respectively) phases compared to BLR. Contrary to sAMS active ascenders which displayed elevated clearances of various metabolites during acclimatization phase, mAMS active ascenders limited metabolite excretion in general, and these alterations tended to be transient. These alterations were mostly unique to mAMS active ascenders compared to NoAMS active ascenders except for 1-methylnicotinamide, trigonelline and choline responses. Urinary levels of 1-methylnicotnamide consistently decreased for NoAMS group during the acclimatization phase while those for trigonelline and citrate were elevated.

### 3.4 Urinary metabolite clearance at BLR and acute mountain sickness severity

**Figure 8:**
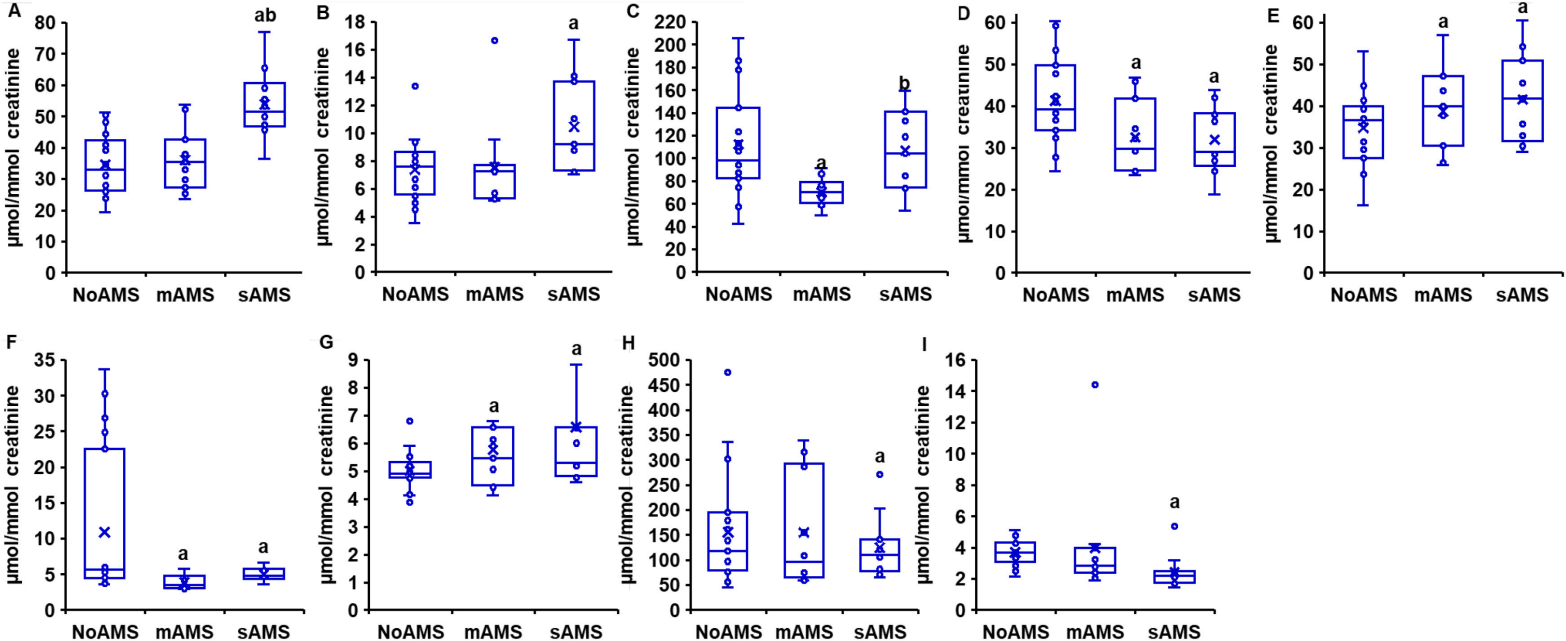
Urinary clearance for **(A)** creatine, **(B)** acetylcarnitine, **(C)** glycine, **(D)** N-methylhistidine, **(E)** 3-methylhistidine, **(F)** 1-methylnicotinamide, **(G)** isobutyrate, **(H)** taurine and **(I)** hypoxanthine at baseline residence (sea level) for sAMS, mAMS and NoAMS subjects. The letters “a” indicates significant differences from NoAMS while the letter “b” denotes significant differences between sAMS and mAMS (*p* ≤ 0.05). NoAMS, no acute mountain sickness; mAMS, mild acute mountain sickness; sAMS, severe acute mountain sickness.

We also examined urine metabolite excretion prior to HA exposure, and how these changes correlate and possibly predict AMS outcome after high altitude exposure. Examination of the urinary metabolite profiles for the AMS groups at BLR indicated that the levels of creatine, ALCAR, N-methylhistidine, 3-methylhistidine, 1-methylnicotinamide, isobutyrate, taurine and hypoxanthine for sAMS subjects were significantly different (*p*≤ 0.05) from those displayed by NoAMS (**Figure 8**, **Table 3**). As shown in **Figure 8A**, averaged creatine clearance levels at BLR for subjects later developing sAMS were elevated by 56% compared to NoAMS/mAMS levels. The ^1^H NMR discovery results were consistent with data generated using an immunoassay (Abcam creatine assay kit, ab65339) (*data not shown*). This group also exhibited significant increases (42% increase) in ALCAR at SL compared to mAMS/NoAMS (**Figure 8B**).

Although sAMS and NoAMS groups displayed similar rates of urinary glycine excretion, mAMS individuals had lower clearance of this amino acid compared to NoAMS and sAMS subjects (34% and 38% decreases, respectively) (**Figure 8C**). Differences in N-methylhistidine urine levels were noted between mAMS, sAMS and NoAMS groups at BLR (**Figure 8D**). sAMS and mAMS subjects decreased N-methylhistidine urinary excretion by 23% and 21%, respectively compared to NoAMS. sAMS and mAMS groups increased 3-methylhistidine excretion (20% and 12% increases, respectively) compared to NoAMS (**Figure 8E**). mAMS and sAMS subjects displayed 54% and 65% decreases in clearance of 1-methylnicotinamide compared to NoAMS individuals (**Figure 8F**). Urinary levels of isobutyrate for both sAMS and mAMS groups at SL increased by 31% and 15%, respectively relative to NoAMS group (**Figure 8G**) while taurine and hypoxanthine levels for sAMS decreased by 25% (**Figure 8H**) and 31% (**Figure 8I**), respectively compared to NoAMS.

### 3.5 Urinary metabolite clearance at high altitude and AMS severity

The data was also examined for changes in the urine metabolite profile between the three AMS outcome groups at HA during both the acute and acclimatization phases. The clearance assessment of the mAMS and sAMS groups were examined relative to that of the NoAMS group. Separate analyses were conducted for the passive and active ascent groups.

Comparing data for mAMS and sAMS passive ascent groups with data for NoAMS passive ascenders highlights the dissimilarities in metabolic responses to hypoxia between susceptible and resistant individuals. In general, mAMS subjects reduced the urinary excretion of various metabolites during their sojourn at HA compared to NoAMS/sAMS subjects (**Figures 2**-**4**; **Table 4**). During the hypoxia acute phase, urinary metabolite clearances for mAMS passive ascenders were clearly distinct from clearances for NoAMS passive ascenders, with 14 metabolites significantly reduced in mAMS vs. NoAMS (**Table 4**). Altered clearances include decreased levels of metabolites classified as (1) energy metabolism by-products such as pyruvate (-49%), lactate (-56%), carnitine (-43%), acetate (-47%) (**Figure 2**; **Table 4**), (2) amino acids and derivatives such as alanine (-58%), glycine (-63%), isobutyrate (-40%) (**Figure 3**; **Table 4**), (3) ketone bodies such as acetoacetate (-51%), β-hydroxybutyrate (-57%), acetone (-42%) (**Figure 3**; **Table 4**), (4) by-products of creatine metabolism such as creatinine (-45%), sarcosine (-35%) (**Figure 4**; **Table 4**) and (5) degradation by-products of other metabolic pathways such as 1-methylnicotinamide (-81%) and choline (-49%) (**Figure 4**; **Table 4**). During this phase, only lactate (28% decrease; **Figure 2B**) hypoxanthine (47% decrease; **Figure 2G**) and 1-methylnicotinamide (73% decrease; **Figure 4D**) were altered in sAMS passive ascent group compared to NoAMS passive ascent group. Urinary pyruvate, lactate, creatinine, alanine, and glycine were also significantly reduced for mAMS passive ascenders relative to sAMS passive ascenders (**Table 4**).

During the acclimatization phase for the passive ascenders, urinary levels of nine metabolites were significantly but transiently altered in mAMS relative to NoAMS (**Table 4**), with most alterations lasting one day. During the acute phase, only β-hydroxybutyrate excretion was reduced in the mAMS passive ascent group but this remained consistently decreased during the acclimatization phase as well. Interestingly, the urinary metabolite response for AMS passive ascenders during the acclimatization period was inverse to that noted in mAMS passive ascenders. Apart from carnitine and choline for which excretion was consistently elevated during the entire acclimatization period, clearance increases noted for seven other metabolites were transient.

Data was also examined for subjects with an additional energy expenditure (active ascent) prior to high altitude exposure to determine how additional energy alterations modify both the urine metabolite pattern and, possibly, AMS outcome. Interestingly, metabolomics data for the active ascenders indicated that differences in metabolite clearances between NoAMS and mAMS or sAMS (**Table 5**; **Figures 5**-**7**) were not as pronounced as those noted for those without the additional energy expenditure (passive ascent group) (**Table 4****;** **Figures 2**-**4**). During the acute phase (HA1), mAMS increased clearance of 6 metabolites relative to NoAMS (**Table 5**; **Figures 5**-**7**). mAMS increased urinary levels of acetate (42%; **Figure 5F**), N-methylhistidine (39%; **Figure 6C**), creatine (53%; **Figure 7A**), creatinine (30%; **Figure 7B**), hippurate and 4-hydroxyphenylpyruvate (86% and 70%, respectively). It is noteworthy that an opposite response was noted when mAMS passive ascent group was compared to NoAMS passive ascent group during this phase as a total of 14 metabolites were reduced (**Table 4**). Also, only urinary taurine was altered for the sAMS active ascent group at HA1 (46% decrease; **Table 5**) compared to NoAMS active ascent group.

During the acclimatization phase for the active ascenders, sAMS displayed more alterations than mAMS (8 vs. 5 metabolites) when compared to NoAMS (**Table 5**). Urinary N-methylhistidine and glycine levels were consistently decreased for mAMS compared to the levels measured for NoAMS group. Most significant changes for sAMS group occurred at HA2 and HA4. All alterations noted for the sAMS such as increases in excretion of amino acids and derivatives (isobutyrate, alanine and glycine), and decreases in clearance of trigonelline, acetate, carnitine, hippurate and 4-hydroxyphenylpyruvate were transient as they were noted only once or twice during the entire stay at HA.

## 4 Discussion

**Figure 9:**
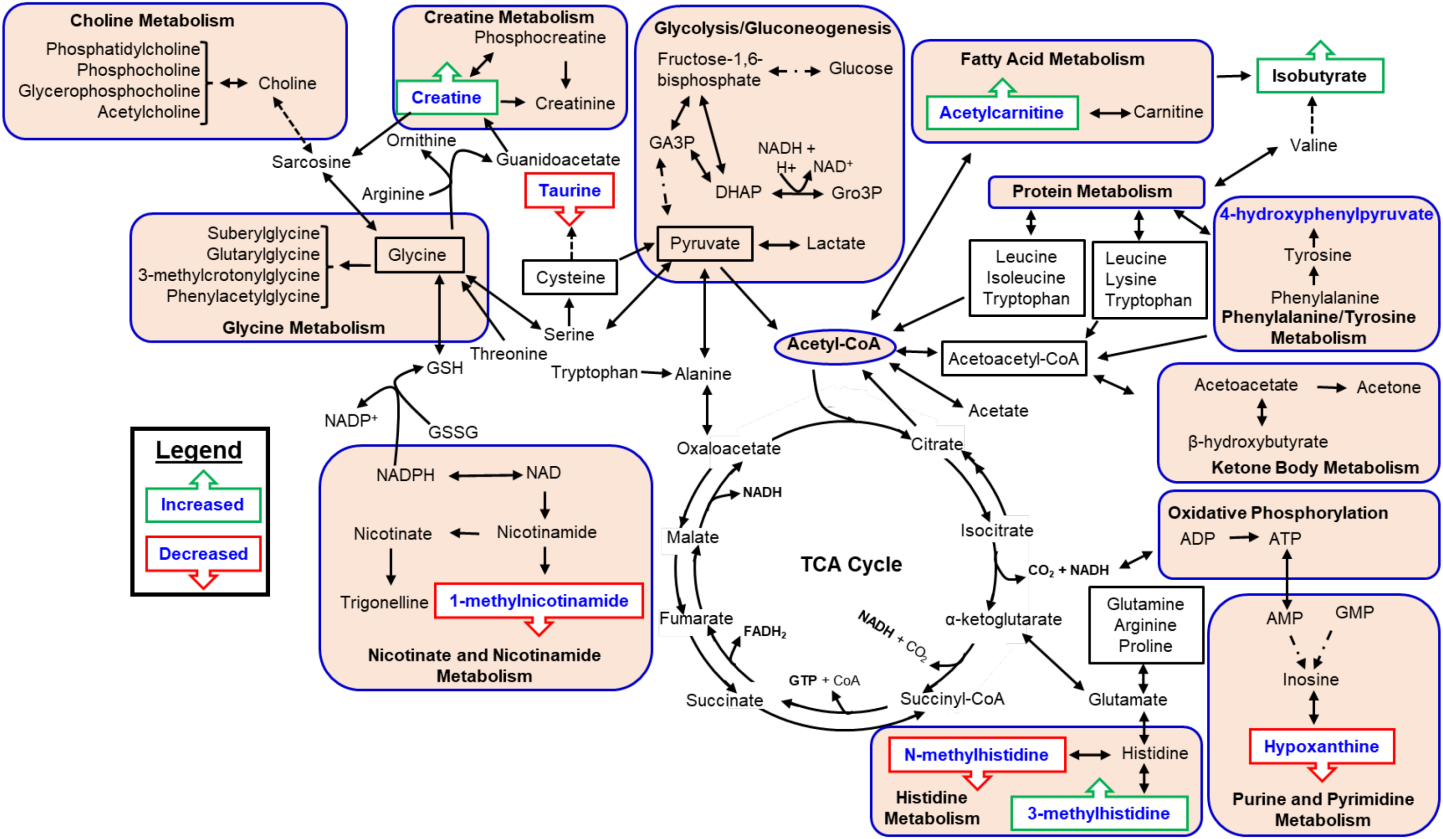
A diagram showing the metabolic pathways for potential urinary metabolite predictors for sAMS and NoAMS subjects at sea level (baseline residence). The diagram was constructed based on the differences in urinary metabolite clearance for sAMS group at sea level relative to NoAMS group. Metabolites shown in green box are those with levels significantly elevated for sAMS group while those shown in red box are those with significantly lower levels for sAMS group (*p*≤ 0.05). Urinary metabolites reported in the previous study (Sibomana et al., 2021) that distinguished NoAMS *vs*. sAMS groups shown in blue font color. ADP, adenosine diphosphate; AMP, adenosine monophosphate; ATP, adenosine triphosphate; FADH2^+^, Flavin adenine dinucleotide (reduced form); GMP, guanosine monophosphate; GSH, glutathione; GSSG, glutathione disulfide; mAMS, mild acute mountain sickness; NAD^+^, nicotinamide adenine dinucleotide (oxidized form); NADH, nicotinamide adenine dinucleotide (reduced form); NADP^+^, nicotinamide adenine dinucleotide phosphate (oxidized form); NADPH, nicotinamide adenine dinucleotide phosphate (reduced form); NoAMS, no acute mountain sickness; sAMS, severe acute mountain sickness; TCA, tricarboxylic acid.

**Figure 10:**
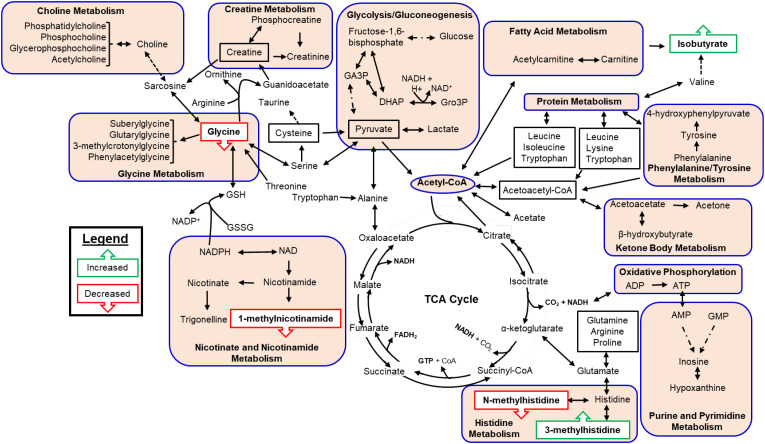
A diagram showing the metabolic pathways for potential urinary metabolite predictors for mAMS and NoAMS subjects at sea level (baseline residence). The diagram was constructed based on the differences in urinary metabolite clearance for mAMS group at sea level relative to NoAMS group. Metabolites shown in green box are those with levels significantly elevated for sAMS group while those shown in red box are those with significantly lower levels for sAMS group (*p*≤ 0.05). ADP, adenosine diphosphate; AMP, adenosine monophosphate; ATP, adenosine triphosphate; FADH2^+^, Flavin adenine dinucleotide (reduced form); GMP, guanosine monophosphate; GSH, glutathione; GSSG, glutathione disulfide; mAMS, mild acute mountain sickness; NAD^+^, nicotinamide adenine dinucleotide (oxidized form); NADH, nicotinamide adenine dinucleotide (reduced form); NADP^+^, nicotinamide adenine dinucleotide phosphate (oxidized form); NADPH, nicotinamide adenine dinucleotide phosphate (reduced form); NoAMS, no acute mountain sickness; sAMS, severe acute mountain sickness; TCA, tricarboxylic acid.

The current study is a follow-on study from a previous investigation (Sibomana et al., 2021) that reported eight urinary metabolites that discriminated AMS susceptible individuals from AMS resistant subjects (NoAMS) prior to altitude exposure. The altered excretion of specific metabolites are the bio-products of creatine, fatty acid, nicotinamide, cysteine, histidine, phenylalanine/tyrosine, purine and pyrimidine metabolisms (**Figure 9**). Of the 8 metabolites identified in the previous study (Sibomana et al., 2021), 7 metabolites classified NoAMS from sAMS in the current study (**Figure 9**), specifically creatine, 1-methylnicotinamide, N-methylhistidine, hypoxanthine, taurine, 3-methylhistidine and ALCAR. Urinary levels for 1-methylnicotinamide, N-methylhistidine and hypoxanthine were significantly lower in sAMS subjects relative to NoAMS individuals, while sAMS subjects demonstrated higher excretion of creatine and ALCAR. The discrepancy between findings from previous studies and our current results may derive from the cut off AMS score used to classify NoAMS individuals. Subjects enrolled in the previous study were only categorized into two AMS classifiers, no/mild AMS and moderate/severe-AMS. Previously, all the subjects with AMS-C scores <1.53 were compared to those with scores ≥ 1.53. Thus, all mild AMS subjects were grouped together with the NoAMS individuals under the NoAMS category, likely skewing the values for the AMS resistant group. In the current study, and unlike most research in predictive AMS markers, mAMS individuals were separated from the NoAMS subjects. All individuals with AMS scores < 0.7 were classified as NoAMS while those with scores ≥ 0.7 but <1.53 were identified as mAMS. Thus, the first study had a larger score range for NoAMS group (scores < 1.53) compared to the current study (scores < 0.7). This new AMS group classification pointed out the dissimilarities in urinary metabolite clearances between mAMS and sAMS subjects at baseline residence (**Figures 9**, **10**). These dissimilarities involve metabolites directly or indirectly associated with energy production such creatine, acetylcarnitine, glycine and metabolite deriving from ATP/GTA degradation such as hypoxanthine (**Figures 9**, **10**). These observations suggest that modulations of energy production pathways prior to ascent may alter mAMS status to sAMS status or vice versa.

### 4.1 Acute mountain sickness incidence rate

The overall AMS incidence rate in the current study for the subjects who were exposed to atmospheric conditions of 3600 masl is 53.7%. This rate is comparable with other rates reported for the same range of altitudes (Maggiorini et al., 1990; Kayser, 1991; Honigman et al., 1993; Murdoch, 1995; Carlsten et al., 2004; Gonggalanzi et al., 2016). The AMS incidence rate among the general population who ascend at 2500 masl is reported to fall between 20% and 25% while the rate for those who climb to altitudes of near 4000 masl is above 40% (Maggiorini et al., 1990; Kayser, 1991; Honigman et al., 1993; Murdoch, 1995; Carlsten et al., 2004; Gonggalanzi et al., 2016). The rate for our previous study when 17 participants were transported to a high altitude of 4300 masl was 64.71% (11 out of 17 subjects) (Sibomana et al., 2021). In general, AMS outcomes at altitudes higher than 4000 masl occur over hours instead of days and the occurrence rates can reach more than 75% (Reeves et al., 1993; Karinen et al., 2008). The development of the altitude-induced sickness is tied to various variables such as the subject’s pre-acclimatization status, history of pre-existing and high-altitude illnesses, elevation of the final altitude, ascent speed, atmospheric pressure, and temperature among others (Hultgren and Marticorena, 1978; Roach et al., 1995; Hultgren et al., 1996; Hackett and Roach, 2001). The ascent speed and the altitude elevation are also tied to severity of the sickness via their impact on the level of hypoxic stress imposed (Maggiorini et al., 1990; Roach et al., 1995; Hackett and Roach, 2001; Gallagher and Hackett, 2004).

The uphill hiking exercise (active ascent) appeared to increase the number of mAMS incidences as the AMS rate for subjects who ascended by foot (passive ascent) to the Kachina peak facility was 33.33% compared to 20% for those transported up to this facility. Roach and colleagues reported an increase in severity and AMS incidence rate among subjects who participated in an active and passive ascents to simulated high altitude (4800 masl) in a hypobaric hypoxia chamber (Roach et al., 2000). The authors noted that 86% of the active ascenders displayed AMS scores of 3 or higher compared with 14% of the passive ascenders who obtained a score of 3. Moreover, AMS symptoms were more pronounced for active ascenders compared to passive ascenders. The authors suggested that the mechanisms responsible for the increase in AMS incidence may have derived from exaggeration of arterial hypoxemia that occurs during exercise. Contrary to the Roach et al. findings showing a rise in AMS incidence severity following a simulated ascent to high altitude, our current study noted that the uphill hiking tended to decrease the rate of the active ascenders who scored in sAMS category (19.05% vs. 35.00% for sAMS active ascent and sAMS passive ascent groups, respectively). The molecular mechanisms behind these observations are unclear but may involve ‘pre-simulation’ of alternative energy pathways that are needed at altitude in the low oxygen environment. However, this hypothesis needs to be further investigated.

### 4.2 Urinary metabolite clearance during acute exposure and acclimatization to high altitude

The results indicated that the urinary metabolite profiles changed significantly as the subjects moved from BLR to HA as well as during their high altitude stay regardless of their ultimate AMS status. This likely reflects the subject’s response to altitude exposure alone (for the passive ascent group, **Table 1**) or to a combination of hiking exercise and altitude exposure (for the active ascent group, **Table 2**). During the acute phase of response to hypoxic conditions, mAMS passive ascenders were more affected by the altitude as they displayed more significant alterations (7 vs. 2 metabolites as noted for NoAMS and sAMS passive ascenders) (**Table 1**). Unexpectedly, these observations suggest the existence of cellular mechanisms designated as an adaptative response to acute exposures to hypoxic conditions that are unique to mAMS subjects. Our data suggest that these mechanisms may involve a reduction of urinary metabolite excretion associated with (1) energy production such as pyruvate and lactate; (2) creatine metabolism such as creatinine and sarcosine; (3) ketone body metabolism (b-hydroxybutyrate); and (4) protein metabolism reflected in amino acids such as alanine and glycine.

Alterations in creatine metabolism may have adverse consequences for skeletal muscles where it is abundantly stored and consumed (Wyss and Kaddurah-Daouk, 2000) and serves as energy storage for high-intensity, short-term exercise (Cooper et al., 2012). Creatine kinase combines creatine with inorganic phosphate to produce the energy source phosphocreatine. Limiting the excretion of creatine degradation by-products under hypoxic environments may signal that creatine is being retained and used as energy storage via its phosphorylated form phosphocreatine (PCr). PCr is utilized when the cellular production of ATP through oxidative phosphorylation mechanisms is reduced due to decreased oxygen availability. Studies have demonstrated that hypoxic conditions are associated with increases in cellular inorganic phosphate contents (Pelosi et al., 1969; Schmidt et al., 1986). Pelosi et al. reported increases of ADP and inorganic phosphate in hypoxic rat hearts along with a decrease in ATP content and oxygen consumption (Pelosi et al., 1969). Increases in inorganic phosphate molecules reported in these studies coupled with a decrease in urinary creatine excretion noted in the current study suggests that, under hypoxia, creatine pools may be channeled towards phosphocreatine synthesis. This assertion is supported by Scott et al. who demonstrated that the rabbit thoracic aorta increased both intracellular free creatine and PCr content by 300% in 4 h when incubated with 40 mM creatine whereas tissue ATP content and PCr/Cr ratio remained unaffected (Scott et al., 1987). Additionally, when the aorta was put in force developing conditions, creatine treated tissue displayed a 3-fold decrease in PCr content compared to untreated rings but no difference in PCr/Cr ratio was noted. A similar pattern of PCr depletion was also noted for creatine treated or untreated contracting muscle exposed to hypoxic conditions. Future efforts will investigate the status of phosphocreatine before and during hypoxia exposures.

Metabolite clearances at high altitude were compared to those at baseline residence. The results indicate that altitude exposure alone (**Table 1**) or together with a combination of hiking to high altitude produced profound alterations in specific metabolite clearance (**Table 2**), reflecting cellular responses to reduced levels of oxygen and hiking-induced increases in energy demand. Most significant alterations in metabolite clearance occurred during the acute phase of response to hypoxic conditions captured after 24 h of HA exposure (HA1), regardless of the AMS status or the means used to reach high altitude (active and passive ascents). Although both groups displayed changes in clearance of various metabolites at HA1, more pronounced alterations were noted in the active ascent group, suggesting that the uphill ascent exacerbated the hypoxia-induced pathway alterations.

Acclimatization is a complex adaptation mechanism that involves molecular and physiological adjustments to protect the cellular functions for survival in hypoxic environments (Chawla and Saxena, 2014; Witt and Huerta-Sánchez, 2019; Storz, 2021). The acclimatization process can take hours, days, weeks, or months depending on the absolute elevation (Chawla and Saxena, 2014). Most urinary alterations seen during the acute phase of hypoxia in this study were transient as they returned to baseline during the acclimatization phase. Although most clearance alterations noted for the passive ascenders during the acclimatization period were transient, regardless of the AMS status, N-methylhistidine elimination for NoAMS passive ascenders was consistently reduced during the altitude exposures while trigonelline excretion displayed a steady increase (**Table 1**). Methylhistidine is released into the blood stream following protein degradation. For instance, skeletal muscle degradation releases 3-methylhistidine which is not reusable in protein synthesis. This has led to the use of its urinary clearance as a suitable biomarker for the rate of skeletal muscle degradation. Thus, reducing the urinary clearance of N-methylhistidine noted in NoAMS subjects may give a hint on possible involvement of protein/amino acid metabolisms as a mechanism used by AMS-resistant individuals to escape the incidence of AMS. Increase in urinary trigonelline noted during acclimatization phase has also been reported in mice urine at 48 h post-hypoxic exposure (Chihanga et al., 2018). Hypoxanthine was a unique metabolite which displayed a steady increase in clearance in sAMS passive ascenders during the acclimatization period. Although elevated levels of hypoxanthine in plasma have been regarded as a hypoxia marker (Saugstad, 1988; Dueland et al., 1991), the current study only noted increased urinary levels in AMS susceptible subjects. Since our study did not measure plasma levels of this metabolite, it is unclear if it was also elevated in the blood for these subjects and/or for NoAMS and mAMS groups. Since urinary levels of this molecule were not altered in both NoAMS and mAMS, it is reasonable to suggest that elevated urinary hypoxanthine levels can be used as a maker of hypoxia for severe AMS susceptible individuals as well.

### 4.3 Cellular levels of creatine and hypoxantine and severity of acute mountain sickness

Of the metabolite alterations seen at sea level for the AMS susceptible subjects, urinary creatine (Cr) clearance was constantly elevated in sAMS relative to NoAMS. Similar results were observed previously (Sibomana et al., 2021), suggesting that urinary Cr clearance may constitute a strong marker for prediction of AMS risk prior to high altitude exposure. Urinary Cr elevation could derive from one or more factors that include a higher dietary intake of Cr-containing foods, a re-absorption deficiency, mutations in its transporters, an upregulation of biosynthesis pathways, a downregulation of its degradation, or a decrease in cellular retention. While dietary Cr intake and supplementation were not controlled in this study, it is unlikely that only AMS susceptible individuals were fed a Cr rich diet or took it as a supplement, thus, eliminating the possibility that increased urinary Cr levels were derived from dietary modulation. While urinary PCr excretion was not examined, urine creatinine and sarcosine levels did not differ between groups. Scot et al. reported that when rabbit thoracic aorta were enriched with Cr and placed under force developing conditions, the Cr-treated aorta displayed a 3-fold decrease in PCr content compared to untreated tissue (Scott et al., 1987). However, no difference in PCr/Cr ratio was noted, suggesting Cr content also declined with a similar decrease rate displayed by PCr. Assuming a similar mechanism in the human body, the Cr increase noted in the current study would also be translated to enhanced PCr level in the body. Increases in cellular PCr would therefore also require changes in Cr kinase activity. Although the current study did not assess the Cr biosynthesis enzymatic activity, there were no significant differences noted in urinary levels of metabolites used by these enzymes as substrates or generated as by-products (such as arginine, glycine, guanidinoacetate). This observation implies that there is no observable upregulation of Cr biosynthesis. Therefore, a higher Cr excretion rate in sAMS subjects most likely derives from a decrease in cellular retention, a re-absorption deficiency, and/or mutations in Cr transporters.

Creatine and PCr are mostly abundant in tissues that require constant or rapid energy supply such as brain, heart, and skeletal muscles (Wyss and Kaddurah-Daouk, 2000; Brosnan and Brosnan, 2007). Cr can be endogenously synthesized in certain tissues or can be obtained from the diet. Cr levels are largely maintained in skeletal muscle, with a concentration of 20-40 mM (Wyss and Kaddurah-Daouk, 2000; Tossenberger et al., 2016). Skeletal muscle contains 95% of Cr content in the body, with the remaining 5% distributed in kidney, testes, brain and liver (Persky and Brazeau, 2001). The majority of Cr in the human body is found as phosphocreatine (PCr), making up to 60% of its stored form while the remaining 40% is in the free Cr form (Cooper et al., 2012). Cr and PCr are also thought to be neuroprotective and may enhance cognitive performance in the brain (Bessman and Geiger, 1981; Marques and Wyse, 2019; Cunnane et al., 2020). The involvement of Cr in improving cognitive performance is supported by data indicating that oral Cr supplementation improves cognitive performance of healthy adults subjected to a hypoxic or normoxic conditions (Rae et al., 2003; Turner et al., 2015). Other studies have suggested that reduced cellular Cr levels increase sensitivity to hypoxic conditions (Wilken et al., 1996; Turner et al., 2015; Scheer et al., 2016). Increased urinary Cr excretion levels suggests a decrease in its cellular retention. Increased Cr elimination noted at BLR for individuals who would become AMS susceptible once exposed to altitude-induced hypoxic conditions suggests existence of a connection between cellular Cr levels and AMS occurrence. Although this connection has not yet been established, our data point to the possibility of a direct or indirect influence of Cr on the susceptibility or resistance to AMS.

Besides Cr, hypoxanthine was also found to discriminate NoAMS and AMS subjects at baseline residence in our previous study (Sibomana et al., 2017). Hypoxanthine is a naturally occurring degradation by-product of ATP and has been regarded as a sensitive indicator of hypoxia (Saugstad, 1988). Under hypoxic conditions, there is an accelerated pattern of AMP breakdown to hypoxanthine. Interestingly, cellular levels of hypoxanthine have been found to correlate with the cellular levels of Cr (Lee et al., 2018). While correlation analyses conducted using the ^1^H NMR urinary Cr and hypoxanthine data for urine samples collected on day -1 (SL) did not indicate a creatine/hypoxanthine correlation for NoAMS individuals (R^2^ = 0.01423; data not shown), the data for sAMS individuals demonstrated a positive relationship between these two metabolites (R^2^ = 0.7255; data not shown). These results agree with a previously published study for which the correlation coefficients for these metabolites were 0.0017 and 0.4309 for NoAMS and AMS groups, respectively (Lee et al., 2018). Lee et al. also reported that hypoxanthine supplementation can reverse hypoxia-induced depletion of cellular Cr and PCr contents although its mechanism of action is not known. Our findings suggest that cellular levels of hypoxanthine may be lower in AMS-susceptible individuals which, in turn, could impair cellular retention of creatine and account for its higher urinary excretion.

### 4.4 Other potential urinary metabolite predictors of acute mountain sickness

Acetylcarnitine (ALCAR) was also among the metabolites that classified sAMS and NoAMS groups at sea level (**Figure 8****,** **Table 3**). ALCAR is synthesized in mitochondria of many tissues by ALCAR transferase enzyme which transfers an acetyl group to carnitine molecule (Farrell et al., 1986; Rebouche, 2004; Jones et al., 2010). ALCAR is critical in cellular energy metabolism as it shuttles acetyl CoA molecules into the mitochondria during fatty acid oxidation. Other research indicates that ALCAR also plays a role in cellular responses to hypoxia-induced stress (Aureli et al., 1994; Barhwal et al., 2007, Barhwal et al., 2009; Scafidi et al., 2010). Barhwal et al. reported that impaired memory retention of rats following exposure to hypobaric hypoxia (7620 masl) was improved by administration of ALCAR during the hypoxic exposure (Barhwal et al., 2009). Findings for that study indicated that daily supplementation of acetylcarnitine to rats during hypoxic exposures ameliorated hypoxia-induced deficits in spatial working memory, oxidative stress, and apoptotic cascades, suggesting that this metabolite plays a significant role in the body’s response to hypoxic stress. Our data identified increased urinary ALCAR clearances in sAMS subjects at BLR, suggesting that preexisting alterations in cellular stores strongly links AMS susceptibility. However, it cannot be ruled out that the occurrence of AMS incidence in these subjects may also be mediated by alteration in energy or lipid metabolism since ALCAR is also required in this cellular process. Further research will clarify this association and mechanism of action.

Our previous study also reported an unidentified metabolite with a singlet at 8.20 ppm that also classified AMS from NoAMS groups. Relative peak intensity for this singlet was significantly elevated for AMS susceptible individuals relative to peak intensity measured for NoAMS individuals. Subsequent investigations successfully identified the peak as belonging to 1-methylnicotinamide proton NMR spectral resonance. The identification of this metabolite was achieved by spiking the urine sample with 1-methylnicotinamide compound and running both 1D and 2D NMR analyses on the sample. The current study identified this metabolite as a strong classifier of sAMS vs. NoAMS as well as mAMS vs. NoAMS (**Table 3**). Both studies found that urinary 1-methylnicotinamide levels were statistically decreased at sea level in AMS susceptible individuals relative to NoAMS individuals. This metabolite is a major by-product of nicotinamide metabolism and has been shown to inhibit the synthesis of nicotinamide adenine dinucleotide NAD^+^ (Hoshino et al., 1984). NAD is a cofactor that plays a crucial role in brain bioenergetics and ATP production (Lautrup et al., 2019). NAD is also one of the key molecules required for cellular glycolysis and the citric acid (TCA) cycle metabolisms. Thus, any alteration in cellular levels of 1-methynicotinamide may have adverse effects on the relationship between this metabolite and NAD. The decreased urinary excretion of 1-methylnicotinamide seen in sAMS and mAMS subjects at sea level (**Table 3**) and on the first day at high altitude (**Table 4**) may reflect an increase in degradation of this metabolite. Unfortunately, our study did not investigate possible correlation of NAD to 1-methylnicotinamide clearance. Published studies have reported that that nicotinamide could have a significant effect on acute hypoxia (Hoshino et al., 1984; Chaplin et al., 1990; Horsman et al., 1990; Chaplin et al., 1991; Horsman et al., 1994), suggesting that altered cellular levels may contribute to hypoxia-induced illness such as AMS. Although no nicotinamide measurements were taken in the current study, data from the urinary 1-methylnicotinamide clearance as well as its degradation by-products noted for AMS susceptible individuals may also reflect cellular nicotinamide status. Thus, altered cellular levels of nicotinamide and/or its breakdown products may also contribute to AMS susceptibility.

Our previous study reported that taurine, 3-methylhistidine, and 4-hydroxyphenylpyruvate (4-HPPA) were also among the metabolite signatures that segregated AMS-susceptible individuals from NoAMS individuals at sea level (Sibomana et al., 2021). The previous findings agree with the current study data which show that the clearances at sea level for these metabolites were statistically different. Data for the previous study indicated that AMS susceptible individuals excreted less taurine at sea level compared to NoAMS subjects. Current results also indicate that sAMS subjects had lower urinary taurine at sea level relative to NoAMS. Of note, various studies have suggested that this biogenic amine plays a significant role in protecting cells against hypoxia-induced damage (Crass and Lombardini, 1977; Franconi et al., 1985; Malcangio et al., 1989; Michalk et al., 1997; Amano et al., 2003; Chen et al., 2009; Chen et al., 2013). Further, under hypoxic conditions, taurine supplementation has been shown to improve cardiovascular function in pigs (Franconi et al., 1985)**,** attenuate vascular remodeling in rats, (Amano et al., 2003)**,** and prevent learning impairment and increase survival time in mice (Malcangio et al., 1989). Although the mechanism of taurine protection against hypoxia-mediated decrements are not well understood, it may act as a potent endogenous agent to induce cellular growth despite oxygen deficiency and improve both osmotic status and calcium homeostasis (Michalk et al., 1997). Collectively, these findings suggest that taurine may play an important role in counteracting hypoxic-induced cellular damage.

In our previous study, we reported an unidentified metabolite with a doublet at 3.33 ppm that also segregated AMS and NoAMS groups. Relative peak intensities for this doublet were statistically higher for AMS susceptible subjects relative to peak intensities measured for NoAMS individuals. Later, the doublet peaks were successfully identified as belonging to 3-methylhistidine spectral resonances. The identification of this metabolite was achieved by spiking the urine sample with 3-methylhistidine compound and running both 1D and 2D NMR analyses on the sample. The urinary levels for this molecule were elevated for sAMS and mAMS subjects at sea level relative to NoAMS, confirming the urinary clearance noted for sAMS in our previous study. The histidine-derived 3-methylhistidine as well as 1-methylhistidine (**Figure 9**) are breakdown bio-products of contractile actin/myosin proteins (Armstrong et al., 2014). Histidine residues in proteins are methylated on their N1 and N3 positions during the post-translational modifications process (Dai et al., 2019; Zheng et al., 2020; Wang et al., 2023). The current data showing that both sAMS and mAMS groups increased 3-methylhistidine clearance while limiting the excretion of 1-methlhistidine (N-methylhistidine) suggest that the histidine methylation enzymes such as SETD3 enzyme that add a methyl group on N3 position of histidine residue in proteins (Dai et al., 2019; Zheng et al., 2020) are more active in sAMS and mAMS subjects than the enzymes that methylate the N1 position of histidine residues, such as the methyltransferase METTL9 (Wang et al., 2023). The data in this current study does not corroborate with previously reported data on 4-HPPA at sea level as a predictor of AMS outcome. Of note, 4HPPA formation derives from the phenylalanine/tyrosine catabolism pathways (**Figure 9**). Phenylalanine and tyrosine levels in the urine were not statistically different between sAMS and NoAMS groups. Since our results are not conclusive about the involvement of 4-HPPA in AMS occurrence, further investigations are warranted.

### 4.5 Mild and severe AMS susceptible individuals respond differently to altitude conditions

Interestingly, the mAMS and sAMS passive ascent groups displayed a distinct and opposite response to hypoxic conditions (**Table 4**), suggesting existence of different cellular mechanisms deployed by the two groups to respond to hypoxia. For instance, mAMS passive ascent group responded to hypoxic conditions by significantly altering clearances of metabolites associated with (1) energy production such as lactate, pyruvate, (2) creatine metabolism such as sarcosine and creatinine, (3) nicotinamide metabolism (1-methylnicotinamide), (4) purine and pyrimidine metabolism (hypoxanthine), (5) amino acids and derivatives (alanine, glycine and isobutyrate), (6) ketone body metabolism (acetoacetate and β-hydroxybutyrate), (7) fatty acid metabolism (carnitine and acetate), and (8) phospholipid and neurotransmitter degradation by-product (choline) compared to sAMS sensitive individuals. All these cellular processes were significantly downregulated for the mAMS passive ascent group. Differences in metabolite clearances between mAMS and sAMS active ascenders were also noted, although these subjects were responding to a combination of altitude-induced hypoxia and increased energy expenditure. This distinct response to altitude may be due to genetic differences existing between the two groups. Very surprisingly, mAMS and sAMS active ascenders (**Table 5**) did not display the distinct differences seen in the mAMS and sAMS passive ascenders (**Table 4**). One possible explanation is the possible masking by the hiking effect (i.e., additional energy expenditure) on cellular metabolisms just prior to energy expenditure shifts required under low oxygen. Unfortunately, the current study did not investigate further why the two groups displayed a distinct response to altitude conditions. However, further studies will be conducted to elucidate the molecular mechanisms by which mAMS and sAMS respond differently to high altitude conditions.

### 4.6 Study limitations and future studies

The current study did not control diet at BLR or HA. Thus, further studies that investigate if dietary factors can influence the individual’s susceptibility or resistance to AMS are needed. The sample sizes were limited to n=9, n=4, and n=7 for NoAMS, mAMS and sAMS passive ascenders, respectively, while the sample sizes for the active ascenders, were n=10, n=7, n=4 for NoAMS, mAMS and sAMS subjects, respectively. Follow-up studies using a larger sample size will be required to validate the findings described here. Only two females were enrolled, and clarification on possible sex differences in both the urinary metabolic profiling at BLR and HA are needed to assess sex-related responsivity and, possibly, unique sex-based metabolite signatures. Follow-up studies are also needed to validate our AMS group classifiers and incorporate them in urinary bioassay prototypes that can be used in predicting AMS risk and outcome severity. Outcomes of these studies are crucial for the development or implementation of targeted individual strategies which attenuate AMS with minimal to no side effects.

## 5 Conclusions

Our previous study identified a set of 8 urinary metabolites that, at BLR prior to HA exposure, segregated subjects who later were susceptible to severe acute mountain sickness upon exposure to hypoxic conditions at high altitude (Sibomana et al., 2021). Results for seven of these metabolites were also found by the current study to be consistent with results reported in the previous study. Differentially expressed metabolites in sAMS group included elevated levels of creatine, acetylcarnitine, 3-methylhistidine and isobutyrate, and decreased levels of N-methylhistidine, hypoxanthine, taurine and 1-methylnicotinamide. Interestingly, pathway analyses examining these metabolites have shown a direct or indirect mechanistic association with pathways involved in energy production with potential influences on physiological responses to hypoxia. The data showing that mAMS passive ascenders displayed more significant urinary metabolite clearance alterations during the acute phase of response to hypoxic conditions compared to NoAMS and sAMS suggest the existence of cellular mechanisms unique to mAMS susceptible individuals that are designated as an adaptative response to acute exposures to hypoxic conditions. We cannot rule out that these mechanisms protect the mAMS from experiencing severe AMS.

In the current study, the diet was not a controlled variable during the time spent at the baseline residence. Subjects and subjects consumed a self-selected diet. As such, further studies are needed to investigate whether diet can influence the individual’s susceptibility or resistance to AMS. The sample sizes were limited to n=9, n=4 and n=7 for NoAMS, mAMS, and sAMS passive ascenders, respectively, while the sample sizes for the active ascenders, were n=10, n=7, n=4 for NoAMS, mAMS, and sAMS subjects, respectively. Follow-up studies using a larger sample size will be required to validate the findings as described here. Data described in the current study are from male subjects only. Clarification on possible gender differences in urinary metabolic profiling at sea level and high altitude are ongoing to allow assessment of gender-related responsivity and, possibly, unique gender-based metabolite signatures for AMS risk.

## Author's note

This manuscript has been classified as “*Distribution A. Approved for public release; distribution unlimited*.” AFMIC/PA approved on 26 August 2025 (Case No. AFRL-2025-3628). The views, opinions, and/or findings contained in this journal article are those of the authors and should not be interpreted as representing the official views or policies, either expressed or implied, of the United States Air Force, the Army, the Department of Defense, the Department of Energy, ORAU/ORISE, or Government. The appearance of commercial trade names or organizations does not constitute an endorsement by the United States Department of Defense (DoD).

## Data Availability

The raw data supporting the conclusions of this article will be made available by the authors upon reasonable request.
